# SERPINA1 drives TACE resistance in hepatocellular carcinoma by competitively binding ITGB3 to block ITCH-mediated ubiquitination and degradation

**DOI:** 10.1007/s13402-025-01155-5

**Published:** 2025-12-30

**Authors:** Liou Zhang, Xiaoxi Bai, Mingyang Du, Wenyue Dou, Ziwen Xie, Jie Liu, Yang Hou

**Affiliations:** 1 Department of Interventional Radiology, Shengjing Hospital of China Medical University, Shenyang, Liaoning Province 110004 China; 2 Department of Ultrasound, Shengjing Hospital of China Medical University, Shenyang, Liaoning Province 110004 China; 3 Department of Radiology, Shengjing Hospital of China Medical University, Shenyang, Liaoning Province 110004 China; 4 Translational Research Experiment Department, Science Experiment Center, China Medical University, Shenyang, Liaoning Province 110122 China

**Keywords:** Hepatocellular carcinoma, Transarterial chemoembolization, SERPINA1, ITGB3, Ubiquitination

## Abstract

**Purpose:**

Transarterial chemoembolization (TACE) is the first-line treatment for intermediate-to-advanced hepatocellular carcinoma (HCC), but post-TACE resistance remains a major clinical challenge. While SERPINA1, a serine protease inhibitor involved in tumor microenvironment regulation, is dysregulated in various cancers, its role in TACE resistance is unclear. This study investigates SERPINA1’s functional and mechanistic involvement in HCC resistance to TACE.

**Methods:**

Serum SERPINA1 levels were measured by ELISA in TACE-treated HCC patients. Functional assays under hypoxic/chemotherapeutic conditions and xenograft models assessed tumor progression. Mechanistic studies integrated qRT-PCR, Western blot, co-immunoprecipitation (Co-IP), GST pull-down, molecular docking, and mass spectrometry to elucidate the SERPINA1-ITGB3 interaction.

**Results:**

Elevated post-TACE serum SERPINA1 levels were significantly associated with poor treatment response and poor prognosis (*P* < 0.05). Functional experiments demonstrated that SERPINA1 promoted HCC cell proliferation, migration, and invasion under hypoxic and chemotherapeutic stress, while xenograft models confirmed its tumorigenic role. Mechanistically, SERPINA1 competitively bound to the EGF-like 2/3 domains of ITGB3 via its C-terminal region (amino acids 320–392), thereby shielding ITGB3 from ITCH-mediated polyubiquitination and proteasomal degradation, which consequently sustained oncogenic signaling pathways.

**Conclusion:**

Our study identifies the SERPINA1-ITGB3 axis as a critical mediator of TACE resistance in HCC, providing a promising therapeutic target to enhance clinical outcomes.

**Supplementary Information:**

The online version contains supplementary material available at 10.1007/s13402-025-01155-5.

## Introduction

Hepatocellular carcinoma (HCC) ranks as the sixth most prevalent malignancy and the fourth leading cause of cancer-related mortality globally, with recent epidemiological studies highlighting its escalating burden [1]. The majority of HCC patients are diagnosed at intermediate or advanced stages due to the insidious nature of early disease, precluding curative surgical intervention and resulting in a dismal 5-year survival rate below 20% [2]. Current clinical guidelines from the American Association for the Study of Liver Diseases (AASLD) and European Association for the Study of the Liver (EASL) designate transarterial chemoembolization (TACE) as the first-line therapy for intermediate-stage HCC [3, 4]. By combining localized chemotherapy with arterial embolization, TACE effectively reduces tumor vascular supply and induces ischemic necrosis [5]. Despite its clinical utility, TACE demonstrates limited efficacy, with over 40% of patients exhibiting primary treatment resistance [6]. Longitudinal analyses reveal concerning survival trends: while 1-year post-TACE survival ranges from 50% to 70%, these rates precipitously decline to 20%-40% at 3 years and 10%-20% at 5 years [7]. This therapeutic shortcoming stems from the palliative nature of TACE, where residual tumor cells frequently persist after incomplete embolization. Clinically, such residual disease correlates with accelerated tumor progression [8, 9]. In HCC patients, the volume of recurrent tumors after TACE increases two-fold in less than 3 months, demonstrating a markedly accelerated growth rate compared to that of primary hepatocellular carcinoma tumors [10]. The molecular mechanisms underlying this aggressive phenotypic shift remain poorly characterized, underscoring the urgent need to elucidate resistance pathways at the cellular and molecular levels.

Emerging evidence implicates Serpin family A member 1 (SERPINA1/α1-antitrypsin), a serine protease inhibitor and acute-phase glycoprotein, as a multifaceted regulator of tumor progression and therapy resistance across malignancies such as pancreatic and lung cancer, primarily through inhibition of apoptosis and promotion of epithelial-mesenchymal transition [11, 12]. While elevated SERPINA1 expression correlates with adverse clinical outcomes in diverse cancers [13], its functional role in HCC, particularly in the context of TACE resistance, remains largely unexplored. This gap is particularly notable given that prior proteomic studies of TACE resistance have predominantly identified dysregulation in hypoxia-inducible factor 1-Alpha (HIF1α) and angiogenic factors [14, 15], seldom implicating a serine protease inhibitor. Of particular significance to post-translational regulation is the ubiquitin-proteasome system (UPS), a pivotal machinery frequently dysregulated in chemoresistance [16]. Within this framework, integrin β3 (ITGB3) emerges as a critical extracellular matrix receptor that orchestrates tumor cell adhesion, survival, and drug resistance in cancers like glioblastoma and breast cancer, often through enhanced survival signaling [17, 18]. A key regulatory mechanism involves ITCH, the E3 ubiquitin ligase responsible for K48-linked polyubiquitination of ITGB3 and its subsequent proteasomal degradation [19]. However, a direct mechanistic link between SERPINA1 and the ubiquitination-dependent regulation of ITGB3 has not been established in any cancer type. Given the established role of UPS in mediating chemotherapy resistance, we hypothesize that in the unique tumor microenvironment of post-TACE HCC, SERPINA1 may confer resistance by a novel mechanism: competitively inhibiting ITCH-mediated ubiquitination of ITGB3 to stabilize it, thereby activating downstream survival pathways-a mechanistic insight that could unveil novel therapeutic vulnerabilities in HCC.

Through integrated clinical and mechanistic investigations, we demonstrate that SERPINA1 promotes TACE resistance in HCC by maintaining elevated serum levels post-treatment and driving tumor aggressiveness under therapeutic stress. Crucially, we reveal that SERPINA1 competitively interacts with ITGB3’s EGF-like domains, preventing its ITCH-mediated ubiquitination and subsequent degradation. These collective findings position the SERPINA1-ITGB3 signaling axis as a promising therapeutic target for combating TACE resistance in HCC.

## Materials and methods

### Clinical samples and TACE procedure

A prospective cohort of 83 treatment-naïve HCC patients was enrolled at Shengjing Hospital between August and December 2023. Eligible patients were adults (18–75 years) with clinically or pathologically confirmed HCC, Child-Pugh A or B liver function, BCLC stage A-C, and an ECOG performance status of 0–2. Patients were excluded if they had received prior antitumor therapy, had extrahepatic malignancies, major organ dysfunction, Child-Pugh C cirrhosis, BCLC stage D disease, or an ECOG ≥ 3.

Pre-TACE assessments included clinical demographics, liver function tests, tumor imaging (contrast-enhanced CT or MRI), and serum AFP levels. TACE procedures were performed by experienced interventional radiologists using digital subtraction angiography. Following femoral artery puncture, superselective catheterization of tumor-feeding arteries was achieved, and embolization was performed using either conventional TACE (idarubicin-lipiodol emulsion with embospheres) or drug-eluting bead TACE (idarubicin-loaded CalliSpheres). Technical success was defined by angiographic stasis and the disappearance of tumor blush. Treatment response was evaluated one month post-TACE using mRECIST criteria. Blood samples for SERPINA1 analysis were collected before and 72 h after TACE. As part of the standard post-procedural clinical workup, routine laboratory parameters, including C-reactive protein (CRP) and liver function tests, were also monitored at these time points. The study was approved by the institutional ethics committee, and all participants provided written informed consent.

### Enzyme-linked immunosorbent assay (ELISA)

Serum SERPINA1 levels were quantified using a commercial Human α1-antitrypsin ELISA Kit (Elabscience, Wuhan, China) according to the manufacturer’ s protocol. Standards and samples were added to pre-coated wells, incubated, and washed, followed by sequential incubation with detection antibody and enzyme conjugate. Absorbance was measured at 450 nm, and concentrations were interpolated from a standard curve. We defined ΔSERPINA1 as the absolute change [SERPINA1(post)-SERPINA1(pre)]; the relative change, denoted %ΔSERPINA1, was calculated as ΔSERPINA1/SERPINA1(pre).

### Establishment of hypoxic and chemotherapeutic cell models

In vitro models combining hypoxia with chemotherapy were established to simulate the post-TACE tumor microenvironment [20–22]. For drug sensitivity assays, cells were seeded in 96-well plates at a density of 10^4^ cells per well and treated with idarubicin hydrochloride (10-1600 nM). After 24 h, cell viability was assessed using a CCK-8 assay, and IC_50_ values were calculated via nonlinear regression. To induce hypoxia, drug-treated cells were transferred to a tri-gas incubator (1% O_2_, 5% CO_2,_ 94% N_2_) for 24 h. Successful hypoxia induction was confirmed by detecting increased HIF-1α protein levels compared to normoxic controls.

### RNA extraction and quantitative reverse transcription PCR (qRT-PCR)

Total RNA was extracted from cells using TRIzol Reagent (Invitrogen) according to the manufacturer’s instructions. RNA concentration and purity (A260/A280 > 1.8) were measured on a NanoDrop spectrophotometer. cDNA was synthesized from 1 µg of total RNA using the GoScript Reverse Transcription System (Promega). Quantitative PCR was performed with GoTaq qPCR Master Mix (Promega) on a StepOnePlus Real-Time PCR System (Applied Biosystems). The reaction conditions were: initial denaturation at 95 °C for 10 min, followed by 40 cycles of 95 °C for 15 s and 60 °C for 1 min. Gene expression levels were normalized to ACTB and calculated using the 2^−ΔΔCq^ method. Primer sequences are provided in Table S1.

### Western blot

Proteins from cells or tissues were extracted using RIPA buffer with protease inhibitors and quantified by BCA assay. After denaturation, equal amounts of protein were separated by SDS-PAGE and transferred to PVDF membranes. The membranes were blocked with 5% BSA and then incubated with primary antibodies at 4 °C overnight, followed by HRP-conjugated secondary antibodies. Protein signals were detected using an ECL substrate, with β-Actin as the loading control. Antibody details are provided in Table S2.

### Cell culture and transfection

The human HCC cell lines Huh-7 and HepG2 were cultured in DMEM supplemented with 10% fetal bovine serum at 37 °C under 5% CO_2_. To regulate SERPINA1 expression, cells were transduced with lentiviral vectors (GenePharma) and selected with puromycin to generate stable cell lines. For ITGB3 manipulation, cells were transfected with either a pEnCMV-ITGB3-3×FLAG overexpression plasmid (Tsingke Biotechnology) using Neofect™ transfection reagent, or with ITGB3-targeting siRNAs (GenePharma) using Lipofectamine 3000. Transfected cells were harvested after 48 h, and transfection efficiency was verified by Western blot. All plasmid and siRNA sequences are provided in the supplementary materials (Fig. S1, S2 and Table S3).

### Cell proliferation, migration, and invasion assays

Cell proliferation was assessed using an EdU-488 kit (Beyotime). Transfected cells were labeled with EdU for 2 h, fixed, and counterstained with DAPI. EdU-positive cells were quantified under a fluorescence microscope.

Cell migration was evaluated using Transwell chambers (8-µm pores, Corning). In total, 5 × 10^4^ cells in serum-free medium were seeded into the upper chamber and incubated for 24 h. Migrated cells on the lower membrane were fixed, stained with crystal violet, and counted from five random fields.

For invasion assays, Transwell chambers were pre-coated with Matrigel (BD Biosciences). Then, 1 × 10^5^ cells were seeded per insert and incubated for 36 h. Invaded cells were stained and quantified as described for the migration assay.

### Animal models

All animal experiments were approved by the Institutional Animal Care and Use Committee of Shengjing Hospital. Twenty-four male BALB/c nude mice (6 weeks old) were acclimatized under specific pathogen-free conditions and randomly divided into four groups (*n* = 6 per group).

Mice received subcutaneous injections of hypoxia/idarubicin-pretreated Huh-7 cells (5 × 10^6^ cells per mouse) in the right axillary region. Tumor volume was measured every 5 days using the formula: length×width²/2. After 30 days, all mice were euthanized, and tumors were collected for further analysis.

### Immunohistochemical staining

Tumor tissues were fixed in 4% paraformaldehyde, embedded in paraffin, and sectioned at 4–5 μm thickness. After deparaffinization and antigen retrieval in citrate buffer, endogenous peroxidase activity was blocked with 3% H_2_O_2_. Sections were incubated with primary antibodies at 4 °C overnight, followed by HRP-conjugated secondary antibodies. Signal was developed using DAB, and nuclei were counterstained with hematoxylin. Antibody details are listed in Table S2.

### HPLC-MS/MS

Protein samples (10 µg) were reduced with 10 mM TCEP, alkylated with 25 mM CAA, and digested overnight with trypsin. Peptides were desalted using a C18 column, eluted with 40% acetonitrile, and lyophilized. Samples were separated using a gradient of 0.1% formic acid in water (phase A) and 0.1% formic acid in 80% acetonitrile (phase B), and analyzed on a Thermo Q Exactive HF-X mass spectrometer in data-dependent acquisition mode. Raw data were processed with Proteome Discoverer software (v.2.4).

### Molecular docking

Molecular docking was performed using the HDOCK online platform (http://hdock.phys.hust.edu.cn/) to validate protein-protein interactions. Protein IDs SERPINA1 (P01009), ITGB3 (P05106) and ITCH (Q96J02) were retrieved from Uniprot and docked. Binding conformations and interaction distances (< 5 Å) were analyzed, and results were visualized using PyMOL (Schrödinger, LLC, New York, USA) for 3D diagrams and LigPlus (University of Edinburgh, Edinburgh, UK) for 2D diagrams.

### Co-immunoprecipitation (Co-IP) and GST pull-down

Co-IP and GST pull-down assays were performed to study protein-protein interactions. For Co-IP, Huh-7 and HepG2 cells were lysed using pre-cooled IP lysis buffer. The supernatant was collected by centrifugation, and protein concentration was measured. For immunoprecipitation, the control group used IgG with protein A/G beads, while the experimental group used the target antibody with beads. After washing, immunoprecipitated complexes were analyzed by Western blot. For GST pull-down, SERPINA1-GST and ITGB3-FLAG proteins were expressed and purified from Escherichia coli. Target proteins were incubated with glutathione resin, and non-specifically bound proteins were removed by washing. Bound complexes were eluted with elution buffer and analyzed to confirm interactions.

### Plasmid transfection and binding assay

HCC cell lines were cultured in 100 mm dishes until reaching 60–80% confluency. The culture medium was replaced with fresh complete medium before co-transfection with the indicated plasmids (Shanghai Qinke Biotechnology Co., Ltd.). Plasmid combinations were diluted in Opti-MEM, mixed with 10 µL of Neofect to form transfection complexes, and incubated at room temperature for 30 min. The complexes were added to the cell culture medium and incubated at 37 °C in a CO_2_ incubator for 48 h. Cells were harvested, and proteins were extracted for immunoprecipitation using HA- or FLAG-tag antibodies to analyze protein-protein interactions.

### CHX and MG132 assays

HCC cell lines were transfected with either SERPINA1-targeting shRNA or its negative control (NC) and cultured for 48 h. For half-life determination, cycloheximide (CHX; final concentration 50 µg/mL) was added to the culture medium at designated time points. To block proteasomal degradation, cells were treated with MG132 (final concentration 10 µM) at specified intervals. At 0, 6, 8, 12, and 24 h post-treatment, cells were washed with PBS, lysed, and subjected to Western blot analysis using anti-ITGB3 antibody to assess protein degradation, with β-actin as the loading control.

### Ubiquitination assay

HCC cell lines were pretreated with 10 µM MG132 (MedChemExpress, Shanghai, China) for 12 h. Following PBS washes, cell lysates were subjected to immunoprecipitation using 1 µg of anti-ITGB3-FLAG antibody to enrich the target protein. Ubiquitinated ITGB3 was analyzed by Western blott using anti-MYC antibody to detect Ub-MYC conjugates.

### Statistical analysis

Statistical analyses were performed using SPSS V.26.0 (SPSS Inc., Armonk, NY, USA), and results were visualized using GraphPad Prism V.8.2 (GraphPad Software Inc., San Diego, CA, USA). Normally distributed data were expressed as mean ± standard deviation (SD). Between-group comparisons were performed using independent sample t-tests or Welch’ s t-test for heteroscedasticity. For multiple group comparisons, one-way ANOVA with post-hoc tests was applied if significant differences were detected. Non-normally distributed data were expressed as median (interquartile range) and analyzed using the Mann-Whitney U-test or Kruskal-Wallis H-test. Correlations for non-normally distributed or ordinal data were assessed using Spearman’s rank correlation. Categorical data were presented as frequencies and percentages and compared using the Chi-square test. Multivariate logistic regression analysis was performed to identify independent predictors of treatment response. Survival analysis was conducted using the Kaplan-Meier method, and differences between groups were assessed by the log-rank test. A *P*-value < 0.05 was considered statistically significant.

## Results

### Elevated post-TACE SERPINA1 levels were associated with treatment failure in HCC patients

Table 1 summarizes the baseline characteristics of the 83 enrolled patients, who were stratified into responders (PR&CR, *n* = 34) and non-responders (PD&SD, *n* = 49). No significant differences were observed in baseline demographics, including age (59.00 ± 7.90 vs. 58.18 ± 8.25 years, *P* = 0.653) or gender distribution (male: 85.29% vs. 83.67%, *P* = 0.842). Regarding biochemical indicators, non-responders showed significantly prolonged prothrombin time (13.29 ± 1.61 vs. 12.26 ± 1.38 s, *P* = 0.003) and higher AST levels (median [IQR]: 68.00 [33.00, 100.50] vs. 44.50 [28.50, 63.75] U/L, *P* = 0.028). Tumor staging revealed a significantly higher proportion of BCLC stage C in non-responders (51.02% vs. 35.29%, *P* = 0.005). Serum ΔSERPINA1 levels were significantly higher in non-responders both in absolute value (588.20 ± 200.95 vs. 411.05 ± 173.79 pg/mL, *P* < 0.001) and relative change (0.32 ± 0.12 vs. 0.22 ± 0.10, *P* < 0.001). No significant differences were identified in AFP levels, tumor number or size, Child-Pugh grade, or other clinical variables. Notably, the change in SERPINA1 levels (ΔSERPINA1) did not correlate with the change in C-reactive protein (ΔCRP; *r*=-0.101, *P* = 0.363), indicating that its post-TACE elevation is not a simple consequence of a non-specific systemic inflammatory response.

**Table 1:** Baseline characteristics of the study population

Variables	Total (*n* = 83)	PR&CR (*n* = 34)	PD&SD (*n* = 49)	*P*
Age, year	58.52 ± 8.07	59.00 ± 7.90	58.18 ± 8.25	0.653
Gender				0.842
Male	70 (84.34)	29 (85.29)	41 (83.67)	
Female	13 (15.66)	5 (14.71)	8 (16.33)	
Surgical method				0.260
c-TACE	66 (79.52)	25 (73.53)	41 (83.67)	
d-TACE	17 (20.48)	9 (26.47)	8 (16.33)	
Virus type				0.686
B	58 (69.88)	22 (64.71)	36 (73.47)	
C	15 (18.07)	7 (20.59)	8 (16.33)	
No	10 (12.05)	5 (14.71)	5 (10.20)	
AFP, µg/L				0.212
≥200	42 (50.60)	20 (58.82)	22 (44.90)	
<200	41 (49.40)	14 (41.18)	27 (55.10)	
Tumor count				0.920
Solitary	20 (24.10)	8 (23.53)	12 (24.49)	
Multiple	63 (75.90)	26 (76.47)	37 (75.51)	
Tumor size, cm				0.573
<5	47 (56.63)	18 (52.94)	29 (59.18)	
≥5	36 (43.37)	16 (47.06)	20 (40.82)	
BCLC stage				**0.005**
A	12 (14.46)	10 (29.41)	2 (4.08)	
B	34 (40.96)	12 (35.29)	22 (44.90)	
C	37 (44.58)	12 (35.29)	25 (51.02)	
Vascular invasion				0.355
No	56 (67.47)	21 (61.76)	35 (71.43)	
Yes	27 (32.53)	13 (38.24)	14 (28.57)	
Ascites				0.833
Yes	60 (72.29)	25 (73.53)	35 (71.43)	
No	23 (27.71)	9 (26.47)	14 (28.57)	
Child-Pugh				0.768
A	76 (91.57)	32 (94.12)	44 (89.80)	
B	7 (8.43)	2 (5.88)	5 (10.20)	
PT, s	12.87 ± 1.60	12.26 ± 1.38	13.29 ± 1.61	**0.003**
Total bilirubin, µmol/L	22.80(14.00,35.70)	19.00(13.30,31.60)	24.20(17.05,39.15)	0.086
Serum albumin, g/L	35.42 ± 5.12	35.50 ± 4.99	35.36 ± 5.26	0.901
AST, U/L	54.00(31.00,88.00)	44.50(28.50,63.75)	68.00(33.00,100.50)	**0.028**
ALT, U/L	44.00(26.00,74.00)	44.00(22.00,74.50)	47.00(26.50,76.00)	0.387
ΔSERPINA1, pg/ml	515.63 ± 208.51	411.05 ± 173.79	588.20 ± 200.95	**< 0.001**
%ΔSERPINA1	0.28 ± 0.12	0.22 ± 0.10	0.32 ± 0.12	**< 0.001**
ΔCRP	44.68(29.28,54.28)	49.54(37.96,54.38)	41.95(21.33,54.47)	0.113

Normally distributed data were expressed as mean ± SD and analyzed using parametric tests. Non-normally distributed data were expressed as median (interquartile range) and analyzed using non-parametric tests. Bold letters represent *P* < 0.05

**Fig. 1:**
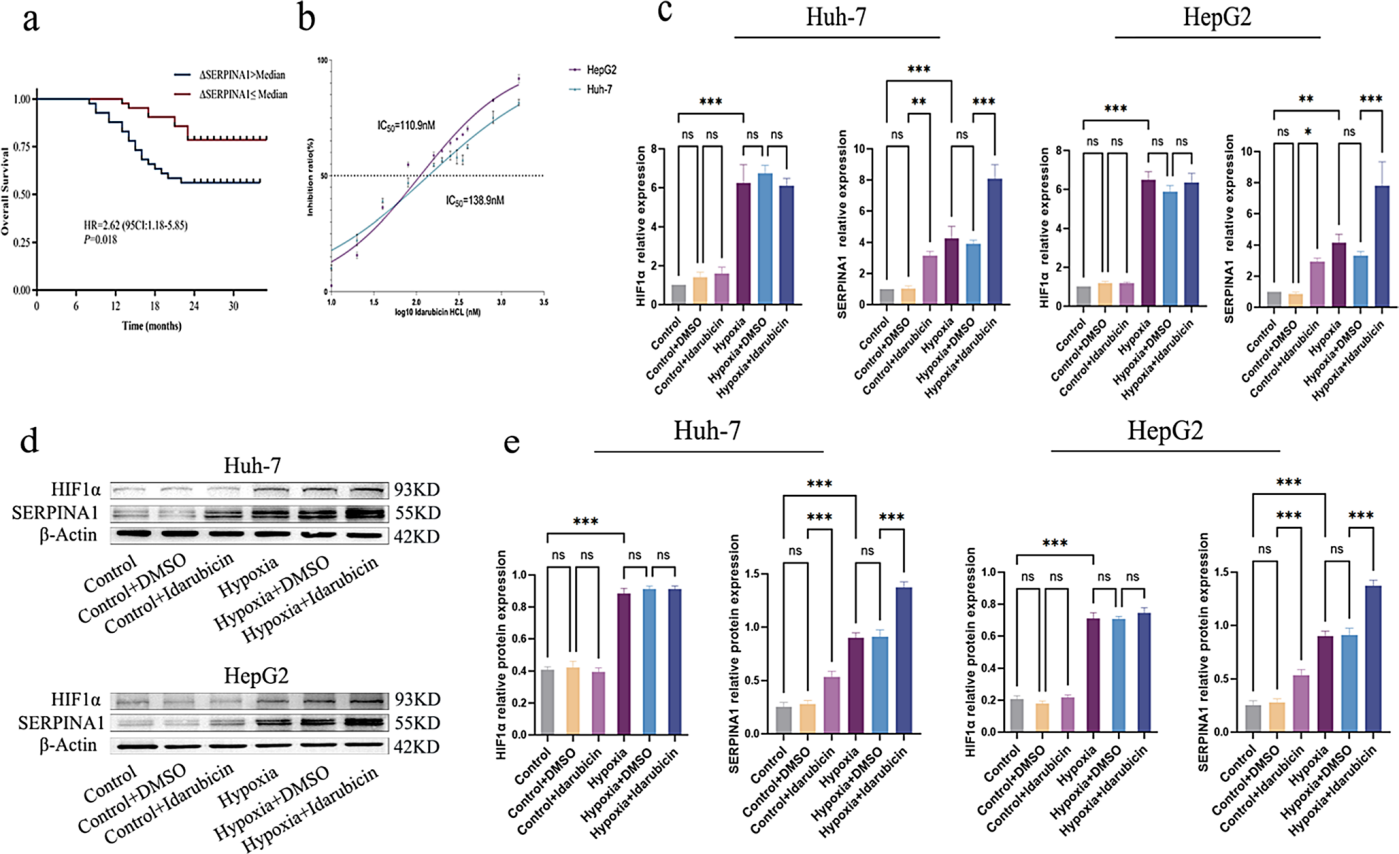
Effects of hypoxia and idarubicin treatment on SERPINA1 and HIF1α expression. (**a**) Elevated serum SERPINA1 predicts inferior OS (HR = 2.62, *P*<0.05) in HCC after TACE. (**b**) IC_50_ values of idarubicin for Huh-7 and HepG2 cells determined by CCK-8 assay. (**c**) qPCR analysis of HIF1α and SERPINA1 mRNA levels in Huh-7 and HepG2 cells. (**d**) Representative Western blot images showing protein expression of HIF-1α and SERPINA1 under different treatments. (**e**) Quantitative analysis of Western blot results for HIF-1α and SERPINA1 protein expression. All quantitative data are presented as the mean ± SD from at least three independent experiments. *P < 0.05, **P < 0.01, ***P < 0.001

Univariate logistic regression identified BCLC stage B (OR = 0.10, 95% CI: 0.02–0.50, *P* = 0.006), stage C (OR = 0.11, 95% CI: 0.02–0.59, *P* = 0.010), ΔSERPINA1 above the median (OR = 0.19, 95% CI: 0.07–0.50, *P* < 0.001), and AST (OR = 0.98, 95% CI: 0.97–0.99, *P* = 0.017) as significant predictors of treatment response. In the multivariate analysis, BCLC stage B (OR = 0.15, 95% CI: 0.02–0.93, *P* = 0.042), stage C (OR = 0.14, 95% CI: 0.02–0.88, *P* = 0.036), and ΔSERPINA1 above the median (OR = 0.19, 95% CI: 0.07–0.55, *P* = 0.002) remained independent predictors, while AST lost statistical significance (OR = 0.99, 95% CI: 0.97–1.01, *P* = 0.224). No significant associations were observed for age, gender, Child-Pugh grade, surgical method, virus type, AFP, ALT, or ΔCRP in either univariate or multivariate analyses. The results of the univariate and multivariate logistic regression analyses are summarized in Table 2. Additionally, elevated serum SERPINA1 levels were significantly associated with poorer overall survival (HR = 2.62, *P* < 0.05) in HCC patients following TACE treatment (Fig. 1a).

**Table 2:** Univariate and multivariate analysis of treatment response factors

Variable	Univariate		Multivariate	
	OR (95%CI)	*P*	OR (95%CI)	*P*
Age	1.01 (0.96 ~ 1.07)	0.649		
Gender				
Male	1.00 (Reference)			
Female	0.88 (0.26 ~ 2.98)	0.842		
Child-Pugh				
A	1.00 (Reference)			
B	0.55 (0.10 ~ 3.02)	0.491		
Surgical method				
c-TACE	1.00 (Reference)			
d-TACE	1.85 (0.63 ~ 5.40)	0.264		
BCLC				
A	1.00 (Reference)		1.00 (Reference)	
B	0.10 (0.02 ~ 0.50)	0.006	0.15 (0.02 ~ 0.93)	0.042
C	0.11 (0.02 ~ 0.59)	0.010	0.14 (0.02 ~ 0.88)	0.036
Virus type				
B	1.00 (Reference)			
C	1.43 (0.46 ~ 4.50)	0.539		
No	1.64 (0.0.43 ~ 6.30)	0.474		
AFP, µg/L				
≥200	1.00 (Reference)			
<200	1.75 (0.72 ~ 4.25)	0.214		
ΔSEP				
≤ Median	1.00 (Reference)		1.00 (Reference)	
> Median	0.19 (0.07 ~ 0.50)	< 0.001	0.19 (0.07 ~ 0.55)	0.002
AST	0.98 (0.97 ~ 0.99)	0.017	0.99 (0.97 ~ 1.01)	0.224
ALT	0.99 (0.98 ~ 1.01)	0.418		
ΔCRP	1.02(0.99 ~ 1.05)	0.066		

### Hypoxia and chemotherapy synergistically upregulate SERPINA1 expression in vitro

To simulate the post-TACE microenvironment, HCC cells were exposed to idarubicin under hypoxic conditions (1% O_2_). The idarubicin concentration was determined based on the IC_50_ value derived from the CCK-8 assay (Fig. 1b). Cells were divided into six treatment groups: Normoxia, Normoxia + DMSO, Normoxia + Idarubicin, Hypoxia, Hypoxia + DMSO, and Hypoxia + Idarubicin. Hypoxia induction was confirmed by upregulated HIF-1α expression. Hypoxic induction was validated through HIF-1α protein upregulation. qPCR and Western blot analysis revealed significant elevation of SERPINA1 expression under hypoxic conditions compared to normoxic controls (*P* < 0.05, Fig. 1c-e). This hypoxic effect was potentiated by idarubicin treatment, with the combined hypoxia-plus-chemotherapy group showing the most substantial SERPINA1 induction.

### SERPINA1 promotes cell proliferation, migration, and invasion under hypoxic and chemotherapeutic conditions

**Fig. 2:**
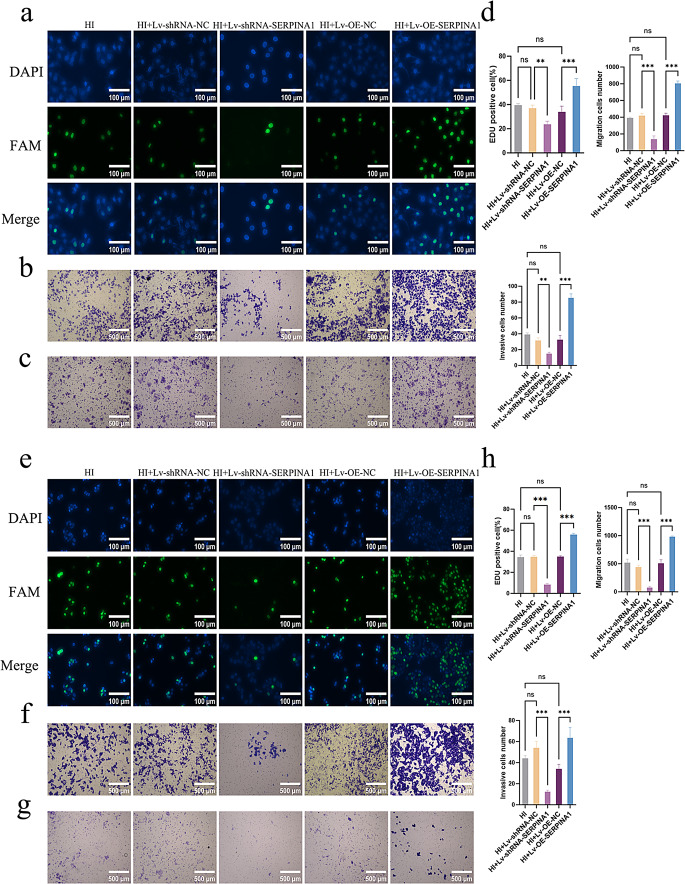
Effects of SERPINA1 upregulation and downregulation on Huh-7 and HepG2 cells proliferation, migration, and invasion under hypoxic and chemotherapeutic conditions. (**a**-**d**) Huh-7 cells: EdU proliferation (**a**), migration (**b**), and invasion (**c**) assays with quantitative analysis (**d**) demonstrate that SERPINA1 overexpression enhances proliferation, migration, and invasion, while its knockdown suppresses these effects. (**e**-**h**) HepG2 cells: Consistent trends are observed in EdU (**e**), migration (**f**), invasion (**g**) assays, and quantification (**h**), confirming SERPINA1’s pro-tumorigenic role. All quantitative data are presented as the mean ± SD from at three independent experiments. *P < 0.05, **P < 0.01, ***P < 0.001

To elucidate the functional significance of SERPINA1 in HCC progression, we established stable SERPINA1-knockdown and -overexpressing cell lines using lentiviral transduction (Fig. S3). Through comprehensive functional characterization under combined hypoxic and chemotherapeutic conditions (HI), we made several critical observations (Fig. 2): First, SERPINA1 overexpression substantially enhanced the malignant phenotype of both Huh-7 and HepG2 cells, as demonstrated by increased proliferative capacity (*P* < 0.05), augmented migratory potential (*P* < 0.05), and heightened invasive properties (*P* < 0.05) compared to vector controls. Conversely, genetic ablation of SERPINA1 expression produced the opposite effect, significantly attenuating these oncogenic behaviors.

### SERPINA1 directly interacts with the EGF-like 2/3 domains of ITGB3

**Fig. 3:**
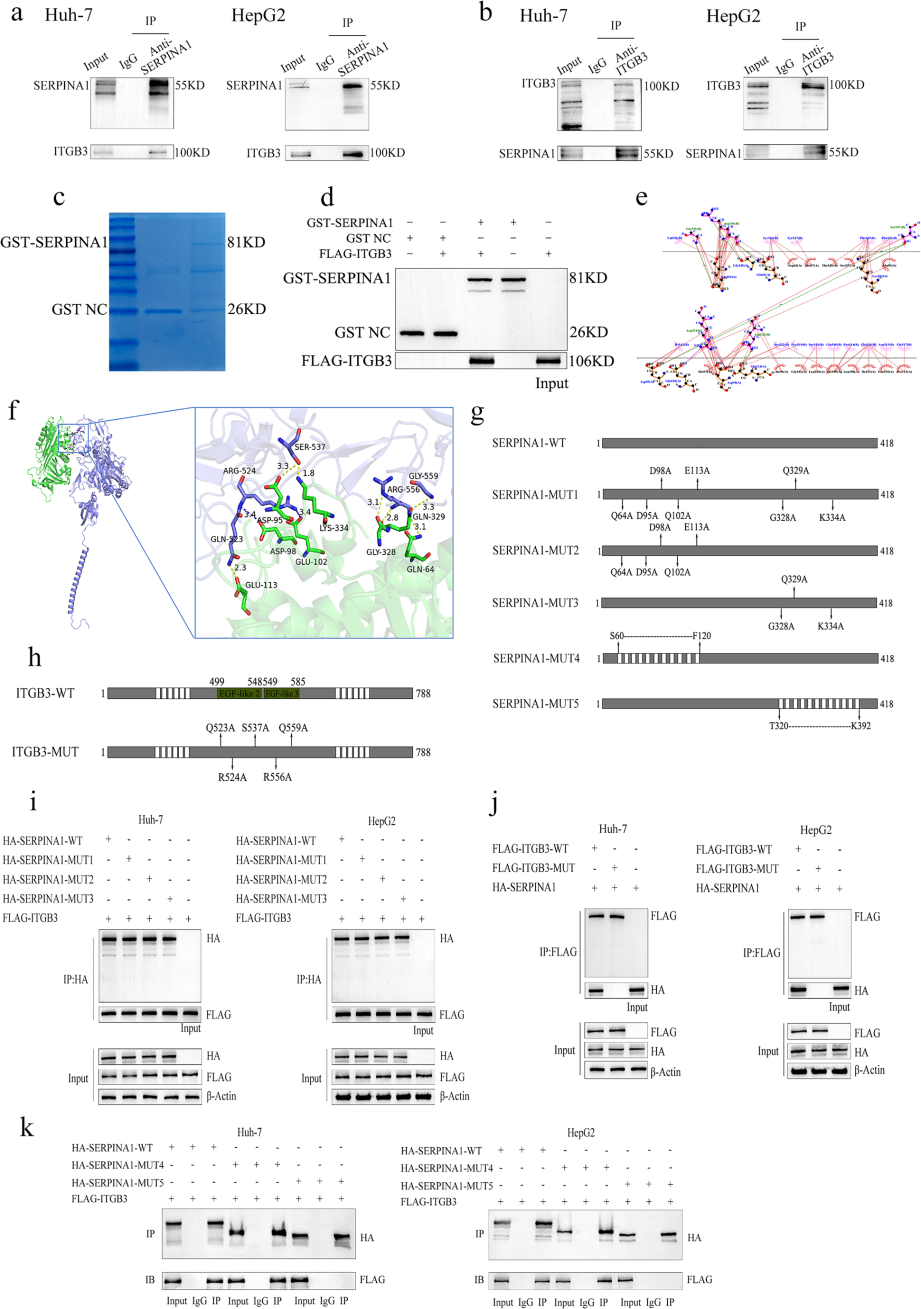
Interaction between SERPINA1 and ITGB3. (**a**-**b**) Co-IP results of SERPINA1 with ITGB3. The Input group served as a positive control, confirming the presence of SERPINA1 and ITGB3, while the IgG group (negative control) showed no bands, ruling out non-specific binding. The anti-SERPINA1 and anti-ITGB3 groups displayed bands for both proteins, confirming their interaction. (**c**) SDS-PAGE detection of GST-SERPINA1 and GST NC proteins expressed in E. coli. (**d**) GST pull-down assay showing FLAG-ITGB3 binding to GST-SERPINA1 or GST NC, validating the direct interaction. (**e**) Two-dimensional molecular docking diagram of SERPINA1 and ITGB3 interaction sites. (**f**) Three-dimensional molecular docking model of SERPINA1 and ITGB3 interaction. (**g**-**h**) Schematic of plasmid constructs for wild-type and mutant SERPINA1 (**g**) and ITGB3 (**h**). (**i**-**k**) Co-IP assays using tag-specific antibodies to validate interaction sites between SERPINA1 and ITGB3

Mass spectrometry analysis of SERPINA1 immunoprecipitates identified ITGB3 as an interacting partner(Fig. S4). Co-IP assays in both Huh-7 and HepG2 cells confirmed this interaction, with reciprocal Co-IP experiments demonstrating robust binding between SERPINA1 and ITGB3 (Fig. 3a, b). To further validate direct binding, GST pull-down assays were performed using purified GST-SERPINA1 and recombinant ITGB3. Western blot analysis confirmed specific precipitation of ITGB3 by GST-SERPINA1 (Fig. 3c, d).

Molecular docking analysis predicted a robust interaction between SERPINA1 and the EGF-like domains 2/3 of ITGB3, with a binding score of -202.03 and a confidence level of 73% (Fig. 3e, f). Site-directed mutagenesis demonstrated that individual mutations at residues 64, 95, 98, 102, 113, 328, 329, and 334 of SERPINA1 had no detectable effect on ITGB3 binding (Fig. 3i). In contrast, ITGB3 mutations at residues 523, 524, 537, 556, and 559 within the EGF-like domains completely abolished binding to SERPINA1 (Fig. 3j). To further characterize the critical binding region, we generated two truncation mutants of SERPINA1: Δ60–120 and Δ320–392. Notably, while deletion of the 60–120 region did not significantly impair binding, the 320–392 truncation completely disrupted ITGB3 interaction (Fig. 3k). These results establish that the C-terminal 320–392 region of SERPINA1 specifically interacts with EGF-like domains 2/3 of ITGB3.

### SERPINA1 competitively binds ITGB3 to block ITCH-mediated ubiquitination and promote malignancy

**Fig. 4:**
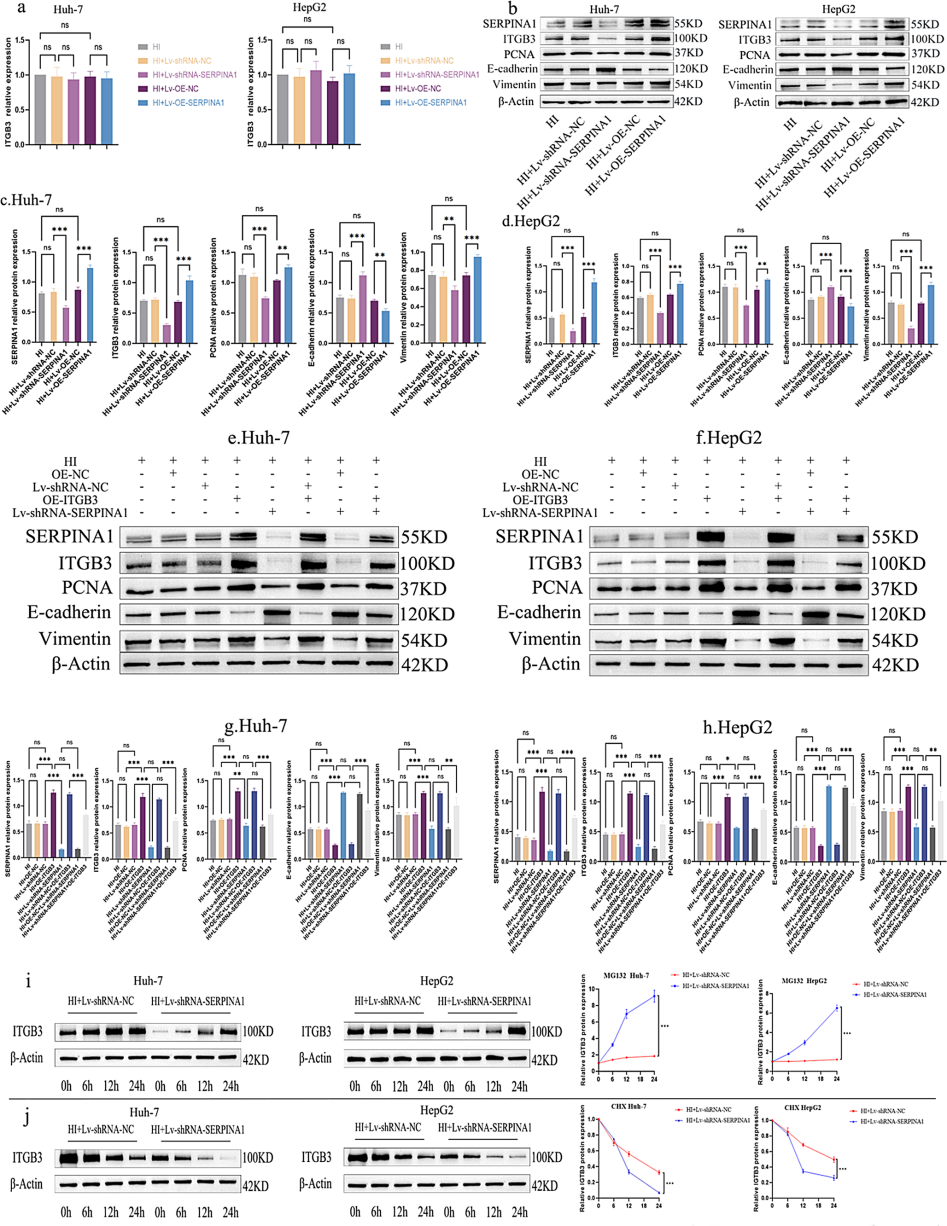
SERPINA1 post-translationally stabilizes ITGB3. HI denotes combined Hypoxia and Idarubicin treatment to mimic the post-TACE microenvironment. SERPINA1 overexpression (Lv-OE-SERPINA1) and knockdown (Lv-shRNA-SERPINA1) were achieved via lentiviral transduction, with empty vector (Lv-OE-NC/Lv-shRNA-NC) serving as their respective controls. (**a**) qPCR analysis showed no significant changes in ITGB3 mRNA levels. (**b**-**d**) Western blot analysis revealed that SERPINA1 post-translationally upregulated ITGB3 protein levels while modulating tumor progression markers. SERPINA1 overexpression increased ITGB3, PCNA and Vimentin expression but decreased E-cadherin, whereas SERPINA1 knockdown showed the opposite effects. (**e**-**h**) Rescue experiments under hypoxia/idarubicin (HI) conditions confirmed SERPINA1’s dependence on ITGB3. (**i**) Proteasome inhibition by MG132 restores ITGB3 levels in SERPINA1-depleted cells. (**j**) CHX chase assays demonstrate SERPINA1 stabilizes ITGB3 by inhibiting proteasomal degradation. All quantitative data are presented as the mean ± SD from at three independent experiments. *P < 0.05, **P < 0.01, ***P < 0.001

While qPCR analysis (Fig. 4a) showed no significant changes in ITGB3 mRNA levels, Western blot analysis (Fig. 4b-d) revealed that SERPINA1 overexpression increased ITGB3 protein levels and knockdown decreased them, indicating post-translational regulation of ITGB3 by SERPINA1. Furthermore, SERPINA1 overexpression increased the expression of PCNA and Vimentin while suppressing E-cadherin (*P* < 0.05) (Fig. 4b-d). Conversely, SERPINA1 knockdown elicited opposing trends, indicating that SERPINA1 may promote tumor progression by modulating ITGB3. Rescue experiments under hypoxia/idarubicin (HI) conditions confirmed SERPINA1’s dependence on ITGB3. ITGB3 expression was experimentally modulated through plasmid transfection for overexpression and siRNA-mediated knockdown for suppression(Fig. S5). In the eight experimental groups, SERPINA1 knockdown reversed ITGB3 overexpression-induced effects, normalizing PCNA/Vimentin elevation and E-cadherin suppression (*P* < 0.05)(Fig. 4e-h), establishing ITGB3 as the primary effector of SERPINA1-mediated tumor progression.

**Fig. 5:**
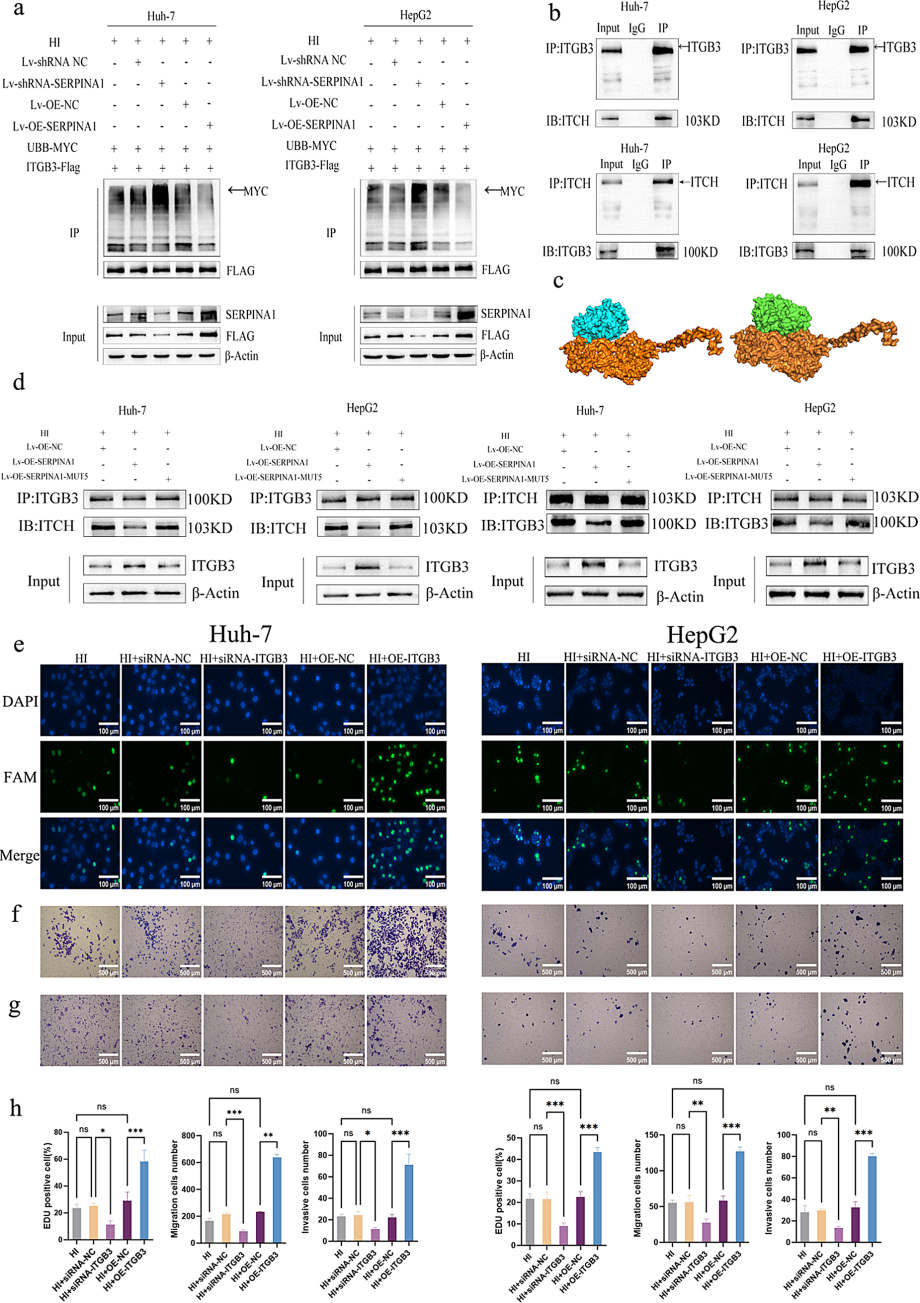
SERPINA1 competitively binds ITGB3 to block ITCH-mediated ubiquitination and promote malignancy. HI denotes combined Hypoxia and Idarubicin treatment to mimic the post-TACE microenvironment. SERPINA1 overexpression (Lv-OE-SERPINA1) and knockdown (Lv-shRNA-SERPINA1) were achieved via lentiviral transduction, with empty vector (Lv-OE-NC/Lv-shRNA-NC) serving as their respective controls. ITGB3 overexpression (OE-ITGB3) was achieved by plasmid transfection, while knockdown (si-ITGB3) was mediated by siRNA, with an empty vector (OE-NC/si-NC) serving as their respective controls. (**a**) Ubiquitination assays demonstrate SERPINA1 negatively regulates ITGB3 polyubiquitination. SERPINA1 knockout enhances while overexpression reduces ubiquitin modification of ITGB3. (**b**) Co-IP assays identify ITCH as the specific E3 ubiquitin ligase binding partner of ITGB3. (**c**) The three-dimensional structural model of ITGB3 (yellow surface representation) demonstrates that both SERPINA1 (blue) and ITCH (green) bind to a hydrophobic binding pocket on the ITGB3 surface. (**d**) Competitive binding: SERPINA1 attenuates ITGB3-ITCH interaction; binding-domain mutation restores ITCH-mediated ubiquitination, confirming SERPINA1 shields ITGB3 from ubiquitin-proteasome degradation.(**e**-**h**) ITGB3 overexpression significantly enhanced proliferation, migration and invasion in both Huh-7 and HepG2 cells, while ITGB3 knockdown produced opposite effects. All quantitative data are presented as the mean ± SD from at three independent experiments. *P < 0.05, **P < 0.01, ***P < 0.001

To investigate the underlying mechanism, we treated cells with the proteasome inhibitor MG132, which restored ITGB3 protein levels in a time-dependent manner, with more pronounced accumulation observed in SERPINA1-depleted cells (Fig. 4i). CHX assays further confirmed that SERPINA1 knockdown significantly shortened ITGB3 protein half-life, demonstrating that SERPINA1 stabilizes ITGB3 by suppressing proteasomal degradation (Fig. 4j). Ubiquitination assays using Ubiquitin B detection revealed that SERPINA1 knockout enhanced ITGB3 polyubiquitination, whereas SERPINA1 overexpression markedly reduced ubiquitin modification (Fig. 5a). Co-IP experiments identified the E3 ubiquitin ligase ITCH as a specific binding partner of ITGB3 (Fig. 5b). Molecular docking analysis revealed competitive binding between SERPINA1 (blue) and ITCH (green) at overlapping epitopes within a hydrophobic pocket of ITGB3’s extracellular domain (yellow) (Fig. 5c). Consistent with these structural predictions, SERPINA1 overexpression competitively disrupted the ITGB3-ITCH interaction (Fig. 5d). Importantly, mutational ablation of the SERPINA1 binding domain restored ITCH-mediated ubiquitination of ITGB3 (Fig. 5d), demonstrating that SERPINA1 directly engages ITGB3 to sterically block ITCH-dependent ubiquitination and subsequent proteasomal targeting.

Finally, to functionally validate ITGB3’s role in HCC progression, we performed phenotypic assays under hypoxia/idarubicin (HI) conditions. As shown in Fig. 5e-h, ITGB3 overexpression significantly enhanced proliferation (EdU assay, *P* < 0.05), migration and invasion (Transwell, *P* < 0.05) in both Huh-7 and HepG2 cells, while ITGB3 knockdown produced opposite effects, conclusively demonstrating ITGB3’s pivotal role in mediating TACE resistance.

### SERPINA1 promotes HCC progression via ITGB3 in vivo

**Fig. 6:**
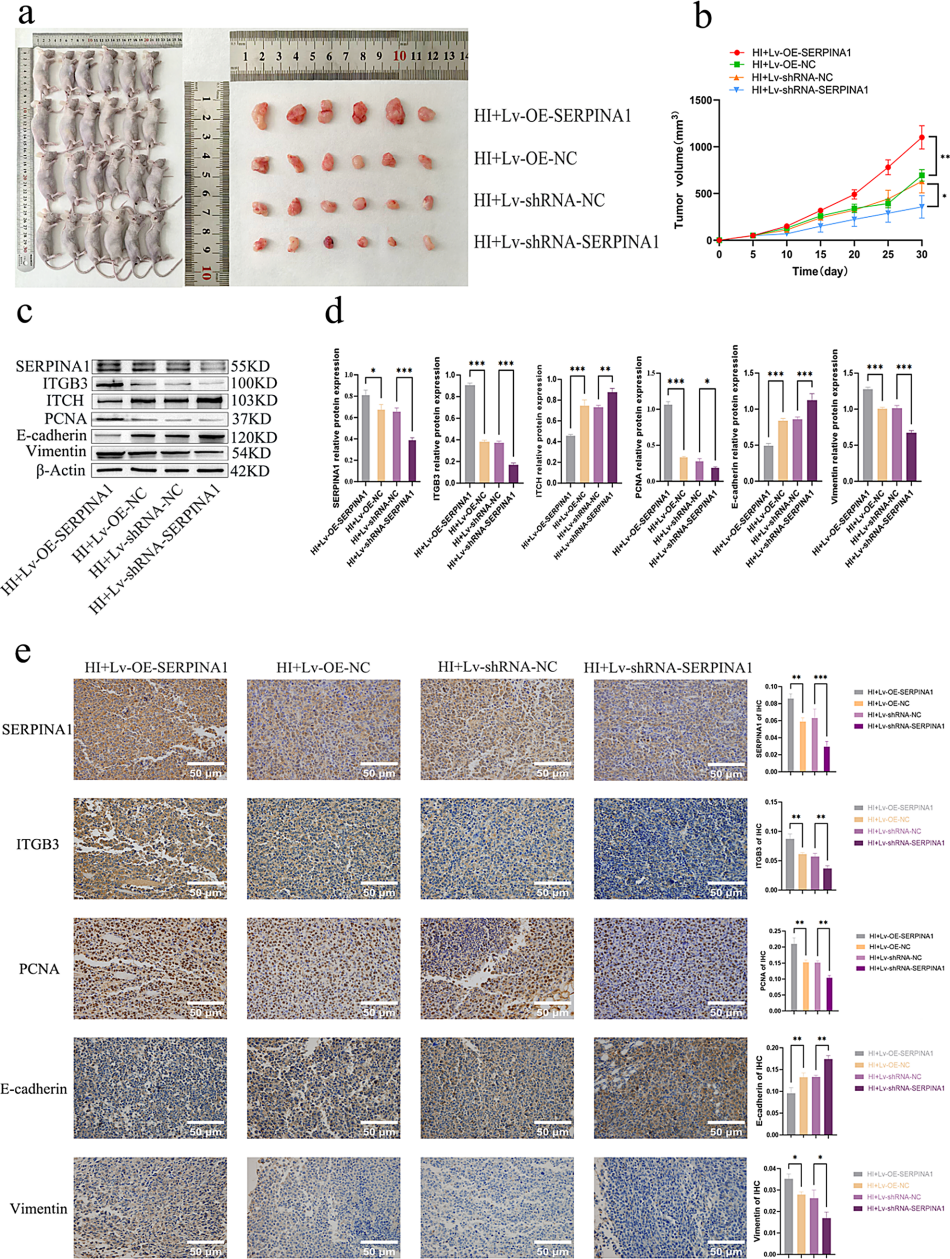
SERPINA1 promotes HCC progression via ITGB3 in vivo. Twenty-four nude mice were randomized into four groups (n = 6) and subcutaneously inoculated with hypoxia/idarubicin-pretreated Huh-7 cells (HI) to establish xenografts. Tumor volume was measured regularly for 30 days before the tumors were harvested. (**a**) Tumor growth in mice: SERPINA1 overexpression promoted tumor growth, while knockdown inhibited it. (**b**) Tumor volume growth curve. (**c**, **d**) Western blot analysis demonstrates that SERPINA1 overexpression upregulates ITGB3 but downregulates ITCH expression, resulting in increased PCNA and Vimentin levels with decreased E-cadherin. These effects were reversed by SERPINA1 knockdown. (**e**) Immunohistochemical staining confirmed SERPINA1’ s effects on PCNA, E-cadherin, and Vimentin expression. All quantitative data are presented as the mean ± SD from at three independent experiments. *P < 0.05, **P < 0.01, ***P < 0.001

In a nude mouse xenograft model of metastatic HCC, Huh-7 cells preconditioned under hypoxic and chemotherapeutic stress were implanted subcutaneously, with SERPINA1 expression genetically modulated via lentiviral transduction. Under hypoxic and chemotherapeutic challenge, SERPINA1 overexpression significantly promoted tumor growth, yielding larger tumor volumes and sizes compared to control (*P* < 0.05). Conversely, genetic ablation of SERPINA1 expression markedly suppressed tumor progression, resulting in significantly reduced tumor volumes relative to control (*P* < 0.05) (Fig. 6a, b). Western blot analysis (Fig. 6c, d) revealed that SERPINA1 overexpression upregulates ITGB3 expression (*P* < 0.05), while downregulating ITCH expression, leading to increased PCNA, and Vimentin levels with concomitant E-cadherin downregulation (*P* < 0.05). These effects were consistently reversed by SERPINA1 knockdown. Immunohistochemistry (Fig. 6e) confirmed these findings, showing significantly increased PCNA + and Vimentin + cell populations with decreased E-cadherin + cells following SERPINA1 overexpression (*P* < 0.05), with opposite effects observed upon SERPINA1 knockdown.

## Discussion

TACE is a widely used treatment for unresectable HCC; however, its efficacy is often limited by resistance development and tumor recurrence. A key contributing factor is the hypoxic microenvironment induced by TACE, which activates HIF1α in residual tumor cells [23]. HIF1α activation drives adaptive responses, including enhanced angiogenesis, increased invasiveness, chemotherapy resistance, and immune evasion, collectively promoting tumor recurrence [15, 24]. Additionally, the local inflammatory response triggered by TACE may lead to immune cell infiltration, paradoxically reducing immune surveillance and fostering a pro-tumorigenic environment [25, 26]. Although the chemotherapeutic agents used in TACE exert cytotoxic effects, they may also induce resistance in residual tumor cells, further compromising therapeutic outcomes. While hypoxia-activated prodrugs (HAPs), such as TPZ and TH-302 [27], have shown early potential in HCC treatment, their safety, efficacy, and patient-specific benefits remain unclear, limiting their clinical application. Therefore, further research is essential to elucidate the molecular mechanisms underlying TACE treatment response, enabling the optimization of therapeutic strategies and improvement of patient outcomes.

SERPINA1 encodes alpha-1 antitrypsin (AAT), a liver-derived serine protease inhibitor that protects tissues from excessive proteolytic activity [28]. As an acute-phase protein, SERPINA1 is induced during inflammatory responses to tissue injury [29]. While this acute-phase nature raises the possibility that its elevated post-TACE levels could partly reflect a general inflammatory response, our finding that its dynamics are uncoupled from CRP levels argues against this being a nonspecific systemic reaction. Instead, several lines of evidence support its specific role in a resistance mechanism. First, the critical finding that elevated SERPINA1 expression correlates with TACE non-response suggests a specific link to treatment failure rather than a universal inflammatory marker. More importantly, our in vitro experiments demonstrated that SERPINA1 expression remains specifically upregulated under the combined hypoxic and chemotherapeutic stress that mimics the post-TACE microenvironment, while many other acute-phase reactants would typically decline after the initial insult. This sustained induction under selective pressure aligns with an adaptive pro-survival function. Furthermore, the mechanistic insight that SERPINA1 competitively inhibits ITCH-mediated ubiquitination of ITGB3 to activate downstream survival pathways provides a direct molecular explanation that transcends a nonspecific inflammatory effect. Given the established link between inflammation and cancer progression [30], elevated SERPINA1 levels may therefore promote tumor resistance and aggressiveness through such specific mechanisms [31]. Indeed, SERPINA1 is overexpressed in multiple malignancies and correlates with increased invasiveness and poor prognosis [32, 33]. In summary, while SERPINA1 elevation post-TACE occurs within an inflammatory context, our data collectively position it not as a passive bystander, but as a key effector molecule that is co-opted by residual tumor cells to drive a specific resistance program against the selective pressures of TACE. A key strength of our study design lies in its multi-faceted approach, which helps disentangle the specific role of SERPINA1 in TACE resistance from a nonspecific acute-phase reaction. First, our clinical cohort analysis established the critical baseline: elevated SERPINA1 levels post-TACE are specifically associated with treatment failure, not merely with the procedure itself. This clinical correlation provided the initial impetus for a mechanistic investigation. To directly address confounding factors like systemic inflammation, we employed controlled in vitro models. By subjecting HCC cells to the precise selective pressures of TACE—namely, combined hypoxia and chemotherapy—in isolation from the complex inflammatory milieu of a whole organism, we could observe the cell-autonomous, adaptive upregulation of SERPINA1. This sustained induction under defined stress conditions in culture provides strong evidence that SERPINA1 is part of a targeted survival response in tumor cells, rather than a passive reflection of systemic inflammation. This foundational finding, derived from a reductionist experimental approach, justified the subsequent investigation into the specific molecular mechanism, which we identified as the SERPINA1-ITGB3 signaling axis.

Our functional characterization of SERPINA1 under hypoxia-chemotherapy combination conditions reveals its non-canonical role as a potent oncogenic driver in HCC progression. The consistent demonstration that SERPINA1 overexpression significantly enhances proliferative capacity, migratory potential, and invasive properties across multiple HCC cell lines (Huh-7 and HepG2) establishes its central role in maintaining malignant phenotypes under therapeutic stress. While our in vitro models specifically recapitulate the dual stress of hypoxia and chemotherapy central to the TACE microenvironment, it is important to acknowledge that they do not encompass the complex immune reactions triggered by local tissue injury in vivo. Nevertheless, our data provide compelling evidence that SERPINA1 upregulation represents a cell-autonomous adaptive survival mechanism, enabling tumor cells to withstand therapeutic stress and enhance their metastatic competence. This cell-intrinsic mechanism likely operates in concert with immune components within the actual TACE tumor microenvironment, a crucial area for future investigation. This observation aligns well with emerging paradigms in cancer biology wherein stress-response proteins are co-opted to drive therapy-induced malignant evolution [34].

To further elucidate the mechanisms by which SERPINA1 regulates downstream signaling, we investigated its potential interaction partners. Mass spectrometry analysis identified ITGB3 as a key interactor of SERPINA1, suggesting that SERPINA1 may modulate HCC cell behavior under hypoxic and chemotherapeutic conditions through ITGB3-mediated signaling. ITGB3, a member of the integrin family, plays a critical role in tumor progression and microenvironment reprogramming [35, 36]. Molecular docking and binding site verification revealed that the EGF-like 2 and EGF-like 3 domains of ITGB3 are essential for its interaction with SERPINA1. To validate these findings in vivo, we established a xenograft model by subcutaneously injecting Huh-7 cells preconditioned under hypoxia and chemotherapy into nude mice. Notably, SERPINA1 overexpression markedly enhanced tumor growth while concurrently increasing ITGB3 expression. Molecular characterization revealed that the SERPINA1-ITGB3 axis modulated the expression of malignancy-associated proteins, including proliferation markers, and EMT regulators, thereby validating our in vitro findings. These data establish that SERPINA1 promotes tumor progression through ITGB3-dependent signaling pathways under therapeutic stress conditions.

This finding not only provides mechanistic insights into the poor efficacy of TACE in HCC but also highlights potential therapeutic targets for intervention. Functionally, our study elucidates a precise competitive binding mechanism wherein SERPINA1 directly competes with the E3 ubiquitin ligase ITCH for binding to the EGF-like domains 2/3 of ITGB3. This competition is particularly significant given ITCH’ s established role in mediating ITGB3 ubiquitination and subsequent proteasomal degradation [19]. Our biochemical evidence demonstrates that SERPINA1 binding effectively blocks ITCH’ s access to ITGB3, thereby preventing its ubiquitination and stabilizing ITGB3 protein levels. This stabilization sustains active integrin signaling pathways that drive oncogenic processes including proliferation, migration, and invasion - a finding consistent with previous reports of ITGB3 stabilization promoting tumor aggressiveness and therapy resistance across multiple cancer types [37]. The implications of these findings extend beyond HCC biology. The SERPINA1-ITGB3 axis may represent a broader mechanism by which stress-responsive proteins hijack integrin signaling to foster therapy-resistant phenotypes. In the context of TACE, where hypoxia and chemotherapy induce SERPINA1 upregulation, our data suggest a feedforward loop in which treatment stress not only selects for SERPINA1-high clones but also potentiates their malignancy through ITGB3 stabilization. This could explain the paradoxical observation that some patients exhibit accelerated disease progression post-TACE.

Our findings also suggest a potential link between genetic SERPINA1 deficiency and TACE response. It could be hypothesized that patients with hereditary alpha-1 antitrypsin deficiency (AATD), who exhibit reduced SERPINA1 expression, may display enhanced sensitivity to TACE. While this clinically relevant prediction remains unexplored—likely due to the rarity of AATD and the complexity of TACE outcomes—our mechanistic data provide a rationale for future validation. Prospective studies comparing TACE efficacy in AATD-associated versus sporadic HCC could substantiate this association and evaluate AATD as a predictive biomarker.

This study has several limitations. First, the single-center, single-ethnicity nature and relatively small size of our clinical cohort limit the generalizability of our findings. The short follow-up period further constrains the analysis of long-term outcomes. Critically, the lack of validation in an independent or multi-ethnic cohort remains a key constraint on the broader applicability of our proposed SERPINA1-ITGB3 model. Additionally, our data do not establish correlations between SERPINA1 and histopathological aggressiveness (e.g., vascular invasion) or include paired ITGB3 immunohistochemistry in patient samples. Second, the animal models incompletely recapitulated the complex tumor microenvironment of clinical TACE. Future studies with large, multi-center, and longitudinally followed cohorts are needed to correlate SERPINA1 dynamics with clinical progression and validate the SERPINA1-ITGB3 axis in human tissues, which is essential for translating these mechanistic insights into biomarkers. Finally, while our mechanistic studies focused on idarubicin to maintain consistency with our clinical cohort, future investigations could explore the relevance of the SERPINA1-ITGB3 axis in the context of other chemotherapeutic agents used in TACE.

In summary, this study elucidates for the first time that the SERPINA1-ITGB3-ITCH regulatory axis serves as a central molecular mechanism underlying TACE resistance in HCC. Specifically, SERPINA1 competitively binds to the EGF-like domains of ITGB3, effectively inhibiting ITCH-mediated ubiquitination and subsequent degradation of ITGB3. This interaction stabilizes ITGB3 protein levels, leading to sustained activation of downstream oncogenic signaling pathways that promote tumor progression. The identification of this axis not only provides a mechanistic explanation for treatment failure in TACE non-responders but also highlights SERPINA1 as both a potential predictive biomarker and a promising therapeutic target.

## Supplementary Information

Below is the link to the electronic supplementary material.

Supplementary Material 1

Supplementary Material 2

## Data Availability

The datasets utilized and/or examined in this study can be obtained from the corresponding author upon reasonable request.
