# Supplementary material for: SERPINA1 drives TACE resistance in hepatocellular carcinoma by competitively binding ITGB3 to block ITCH-mediated ubiquitination and degradation
Source: Cell Oncol (Dordr). 2025 Dec 30;49(1):11. doi: 10.1007/s13402-025-01155-5 (PMC12753576; doi:10.1007/s13402-025-01155-5)
Supplement: Supplementary file 1 — Supplementary Material 1 [file 13402_2025_1155_MOESM1_ESM.docx]

**Supplementary Information（full uncropped Western blots images）**

**Figure 1d**

Huh7


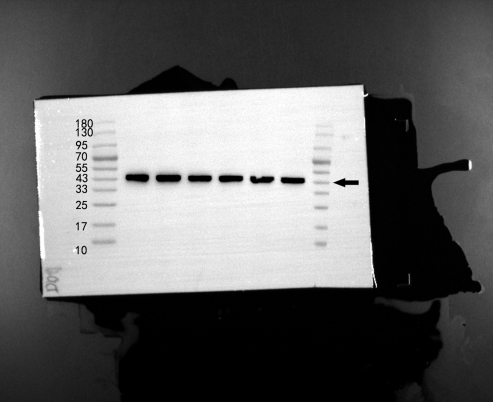

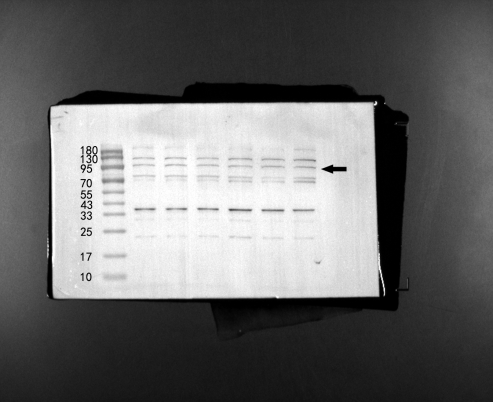

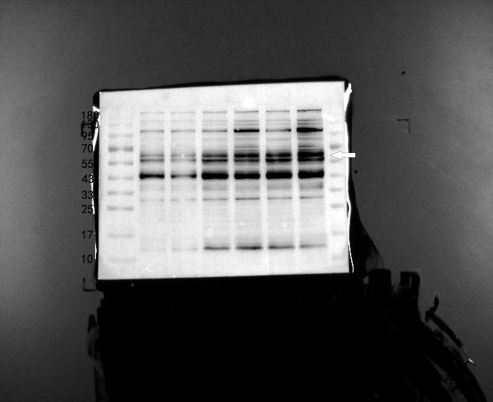


HepG2


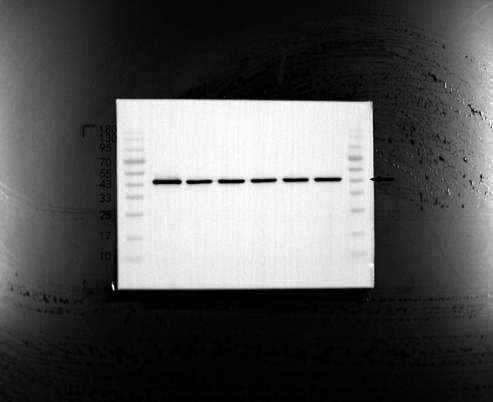

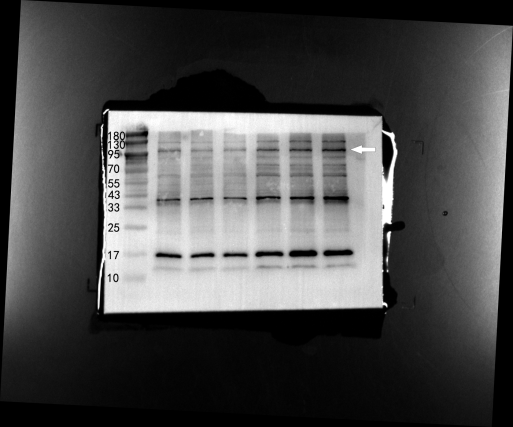

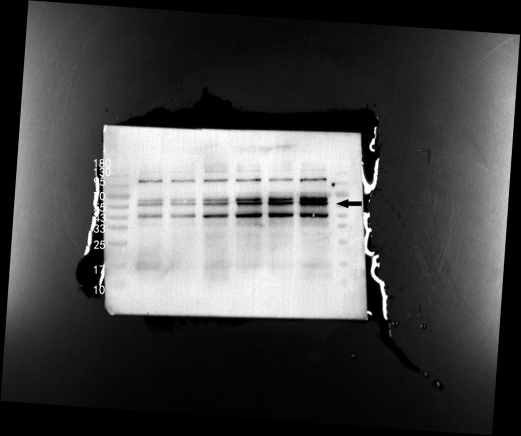


**Figure 3a**

Huh7

**
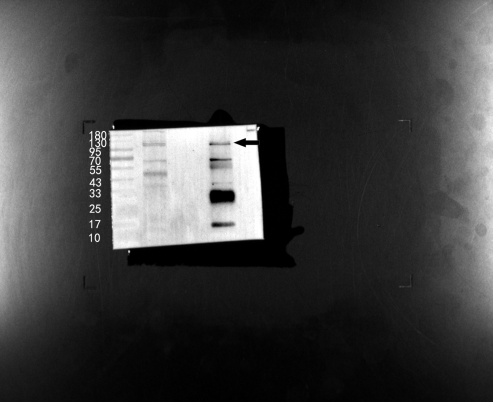

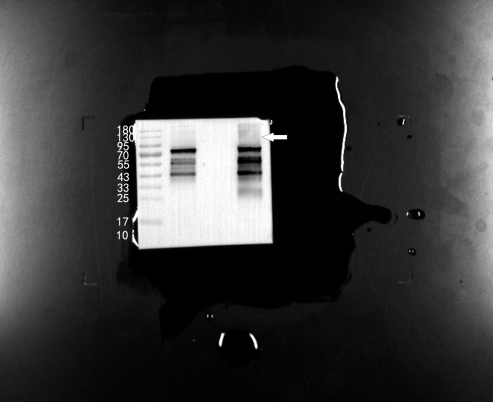
**

HepG2

**
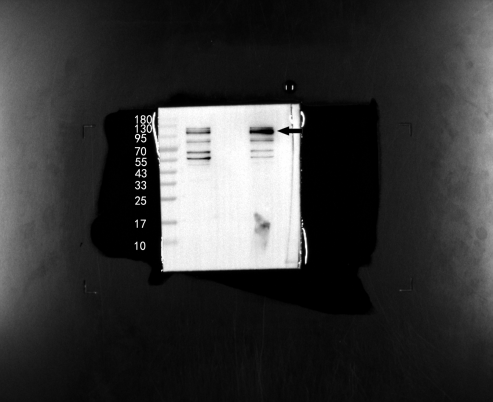

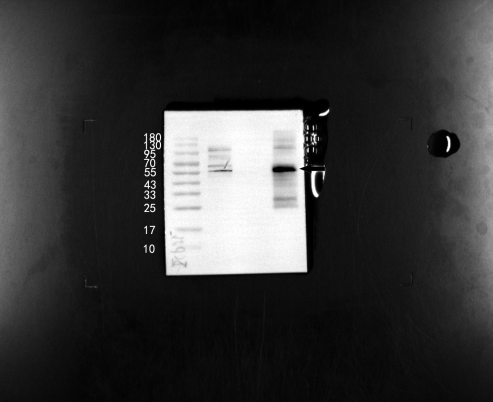
**

**Figure 3b**

Huh7

**
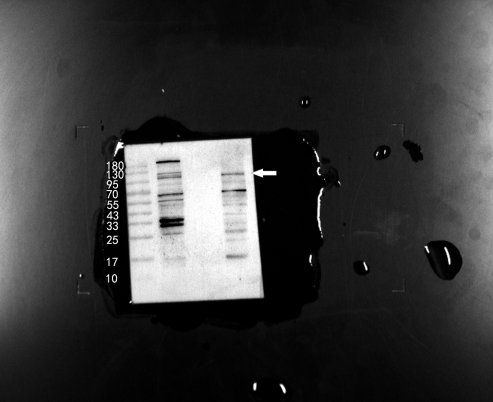

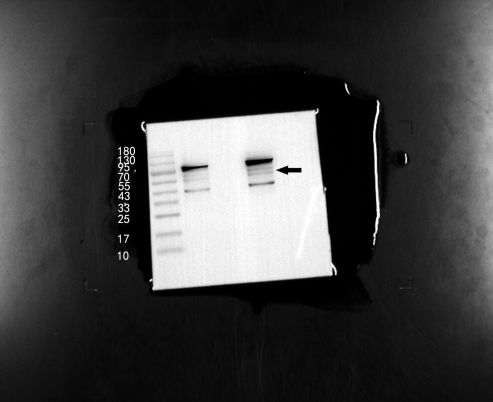
**

HepG2

**
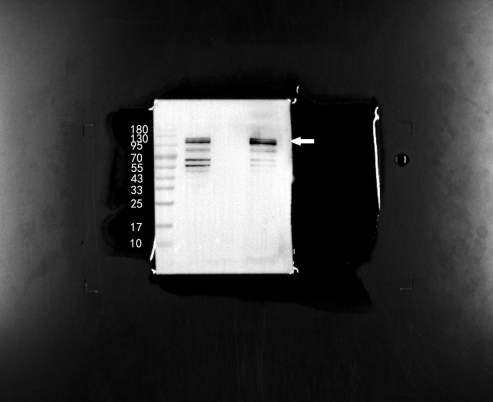

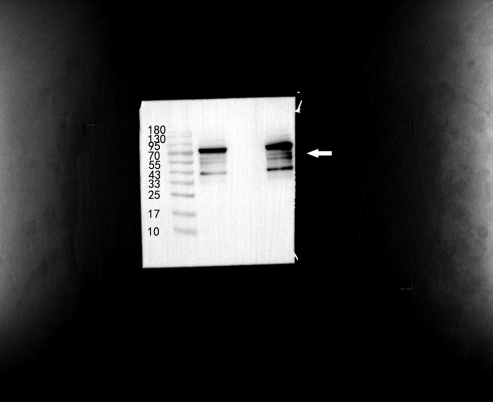
**

**Figure 3c**

**
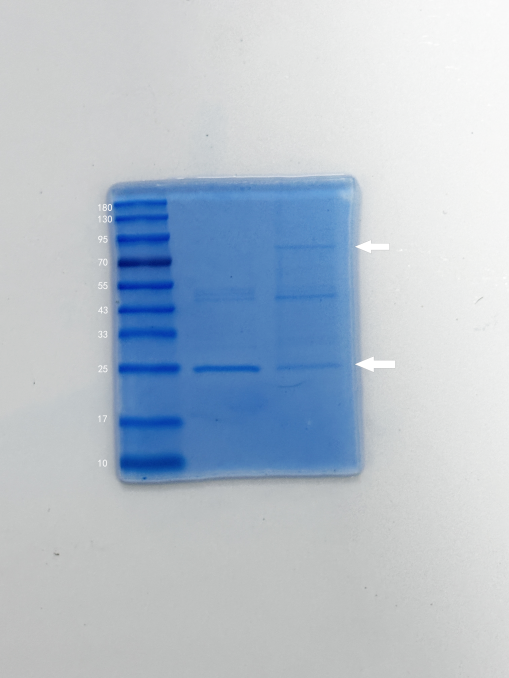
**

**Figure 3d**

**
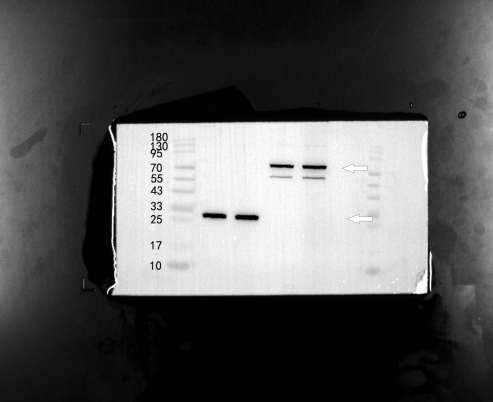

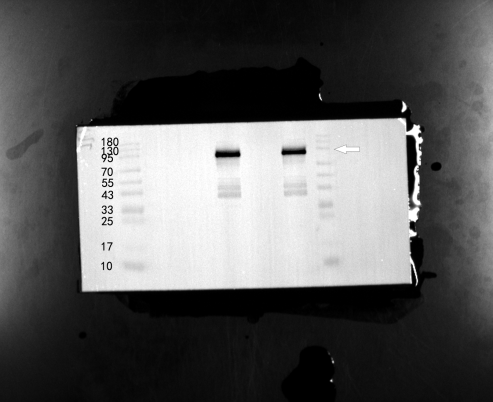
**

**Figure 3i**

Huh7


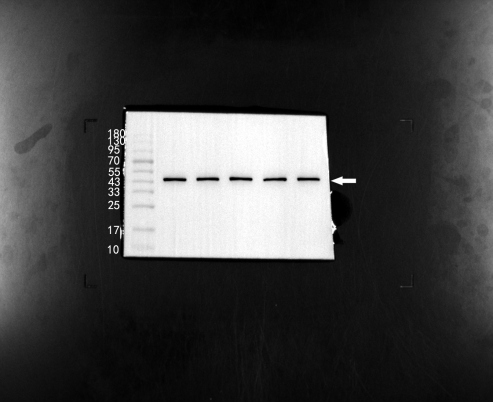

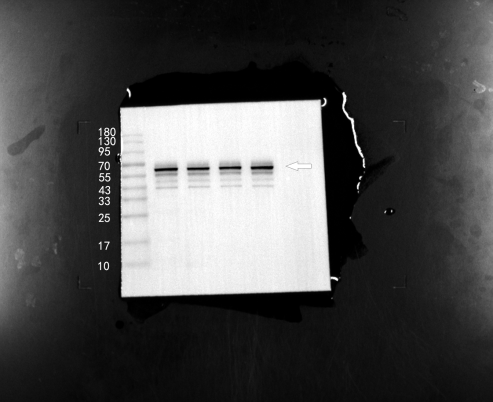

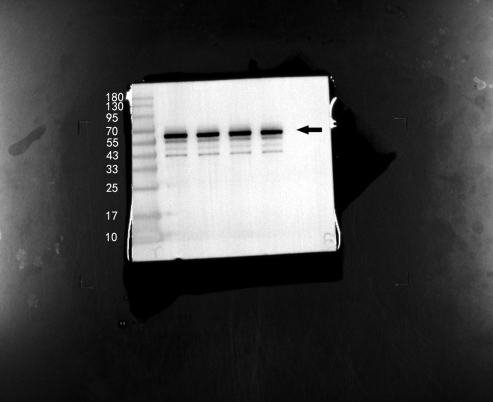

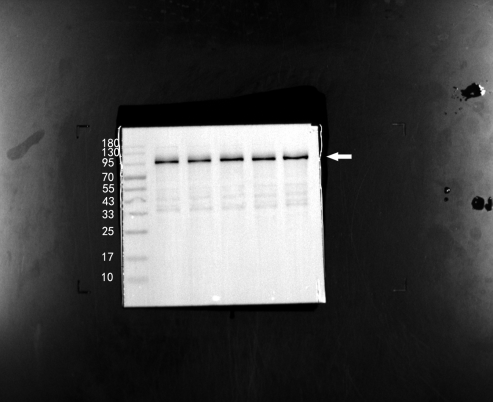

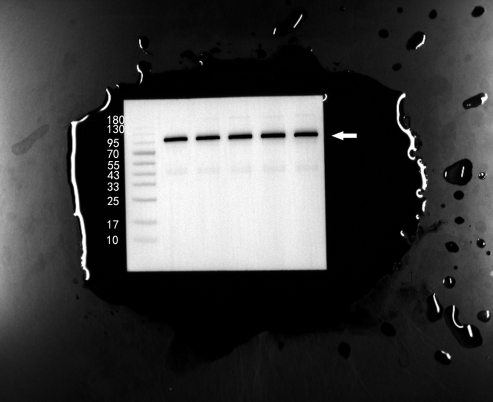


HepG2


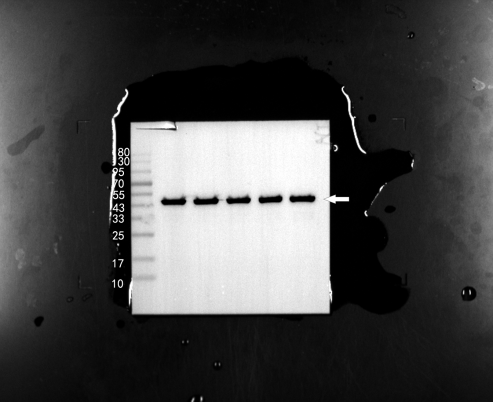

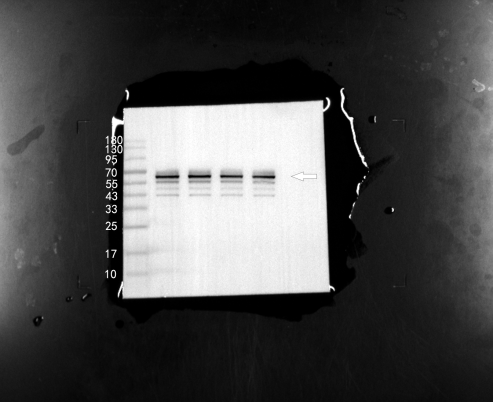

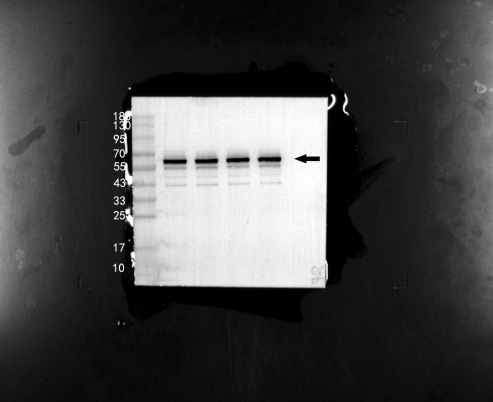

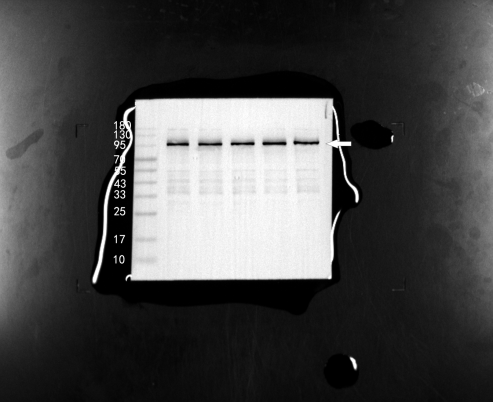

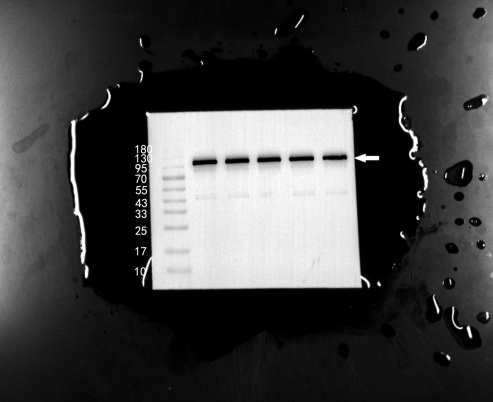


**Figure 3j**

Huh7


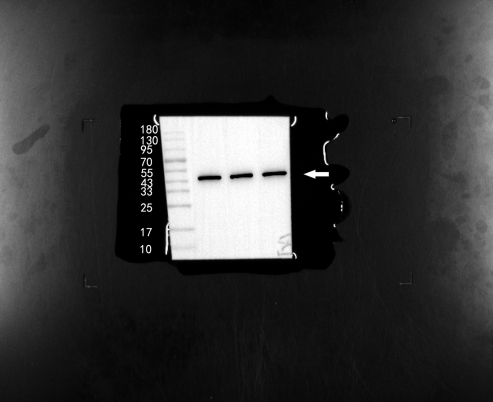

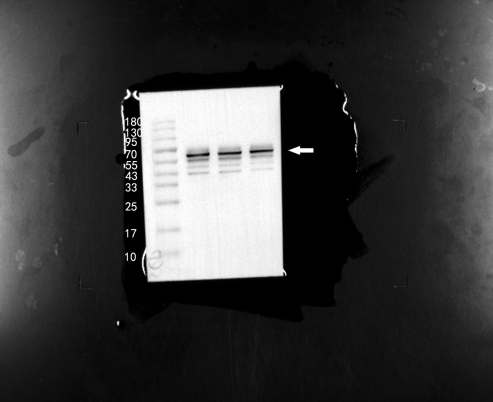

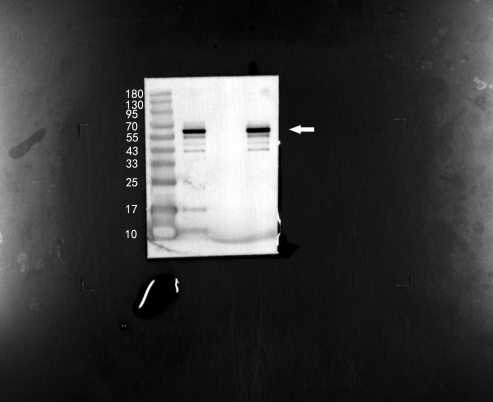

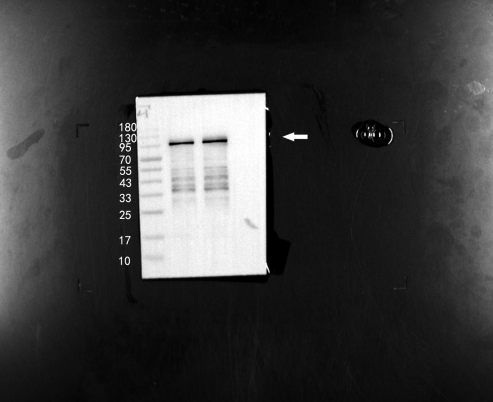

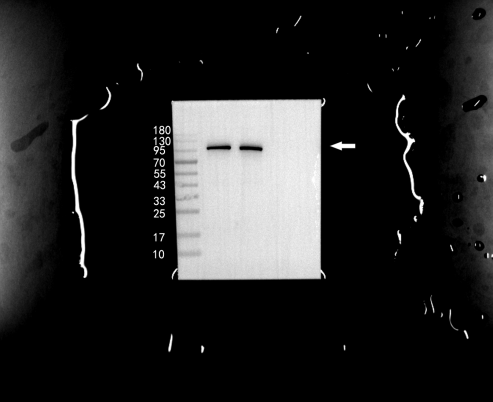


HepG2


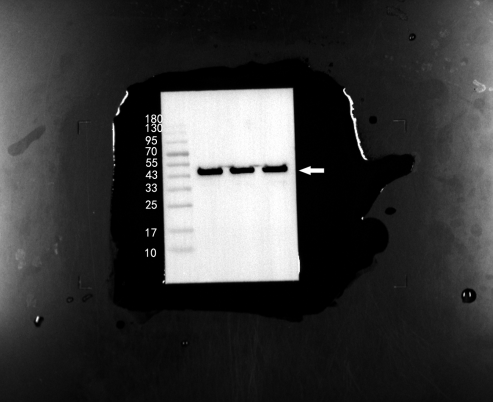

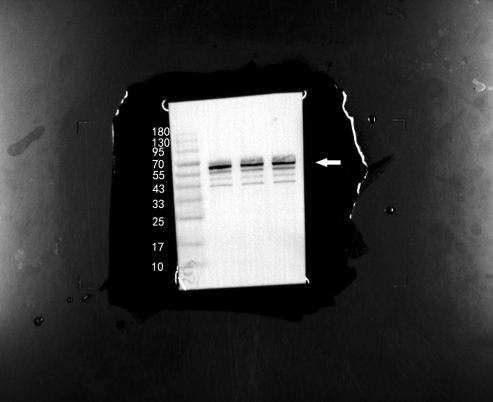

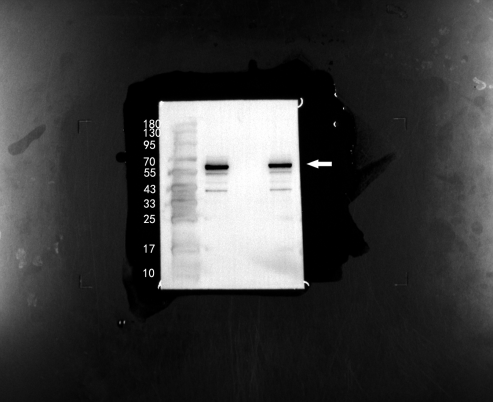

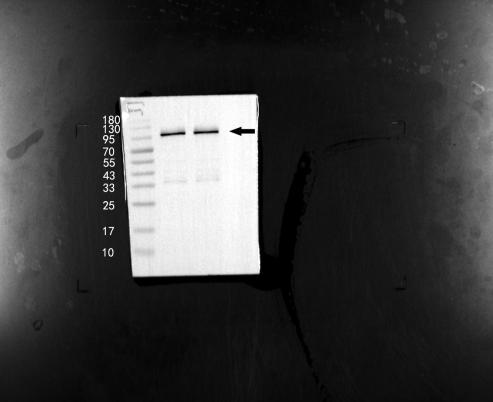

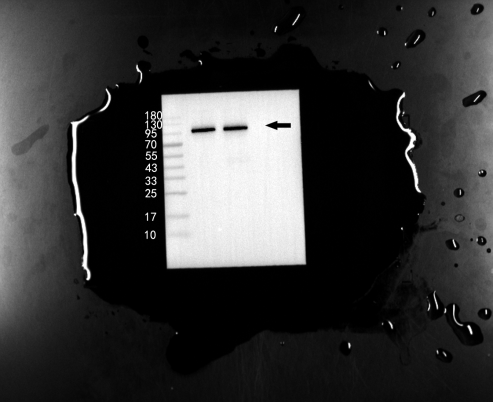


**Figure 3k**

Huh7


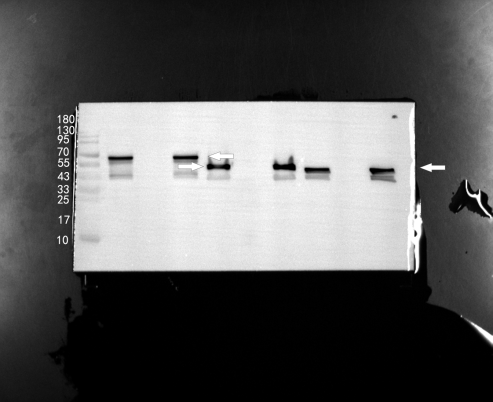

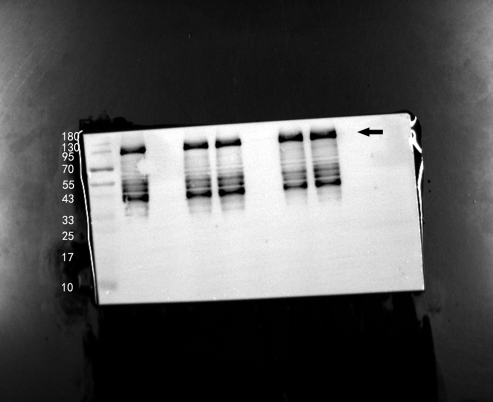


HepG2


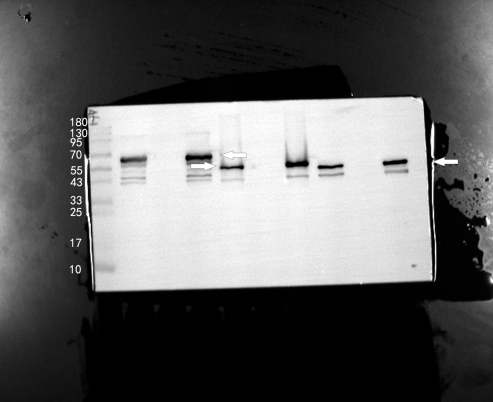

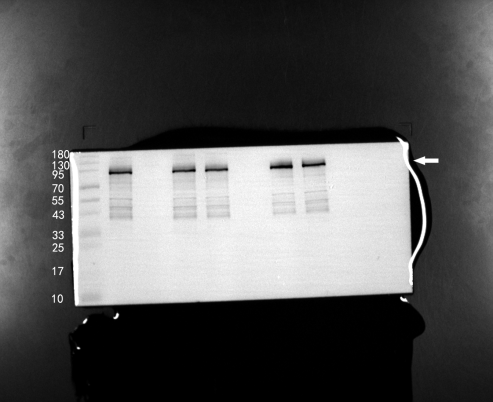


**Figure 4b**

Huh7


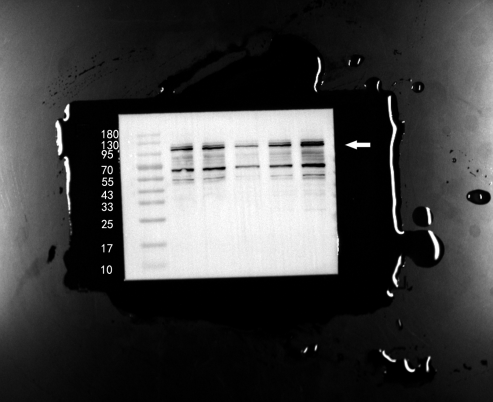

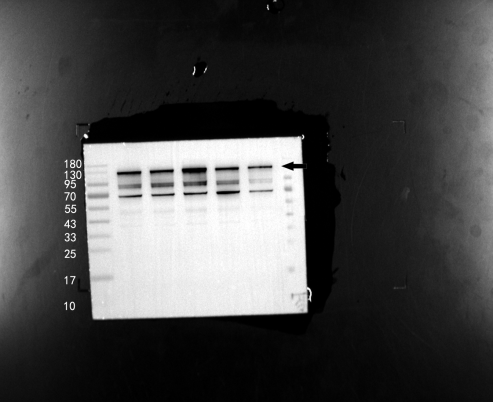

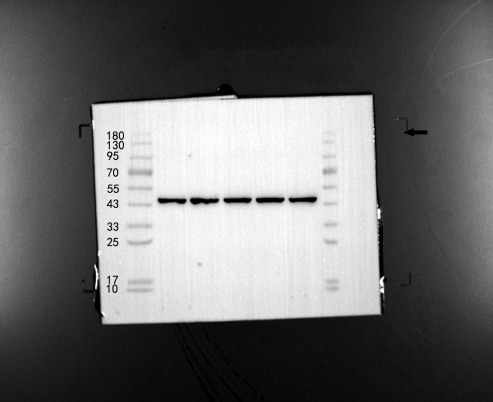

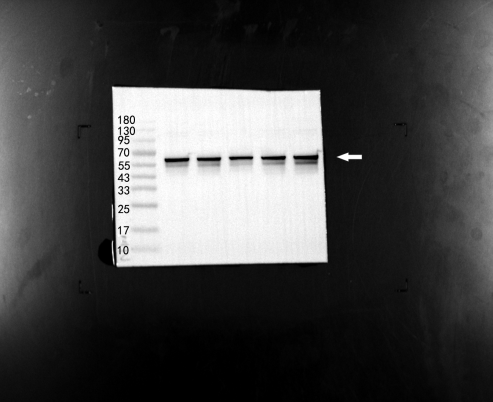

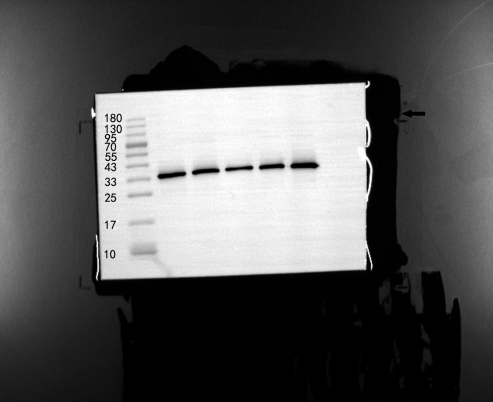

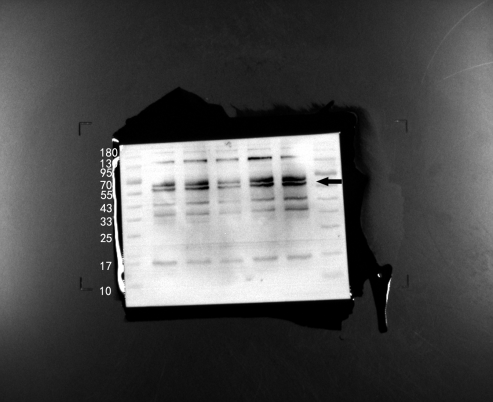


HepG2


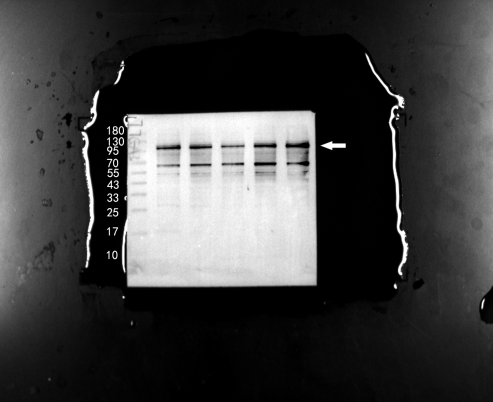

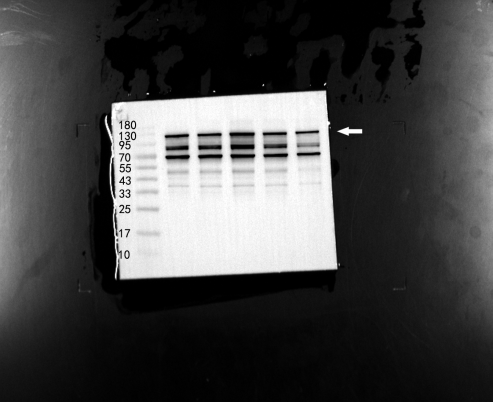

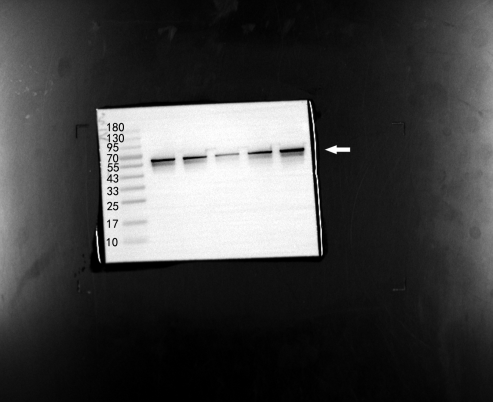

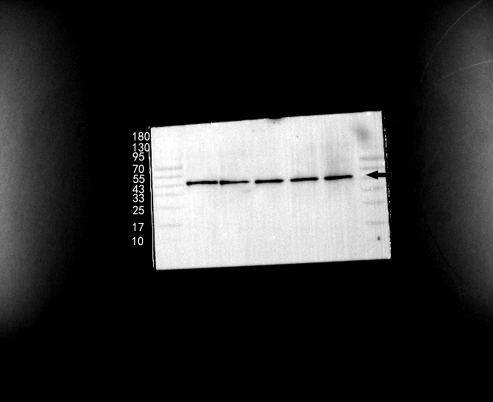

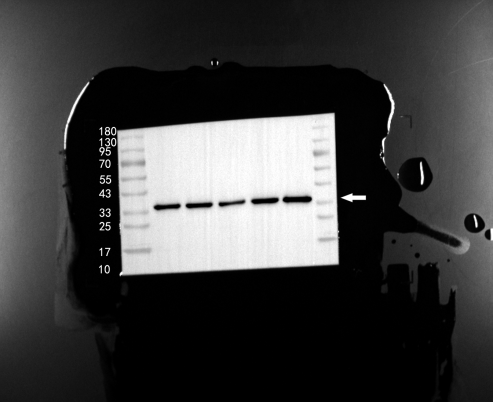

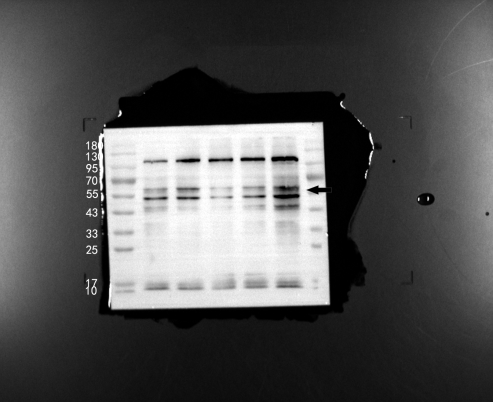


**Figure 4e**

Huh-7

**
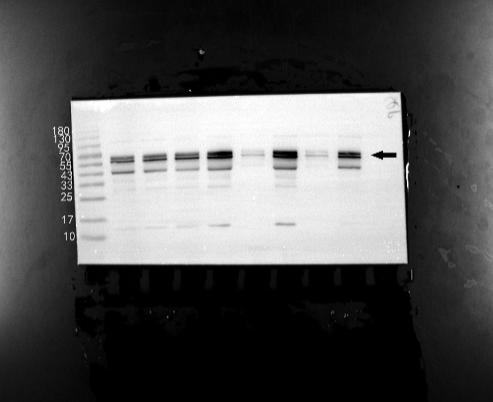

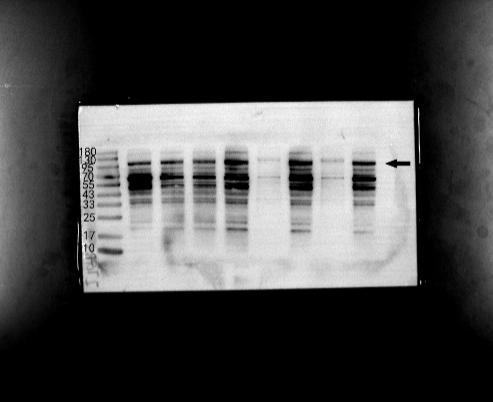
**

**
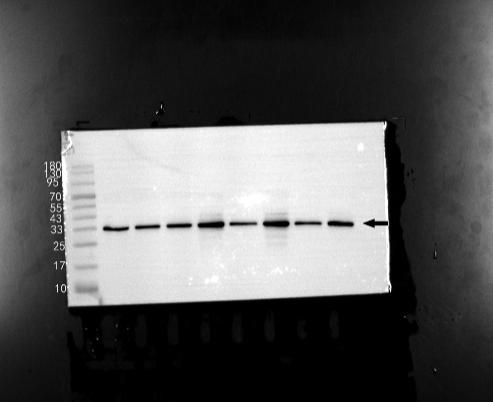

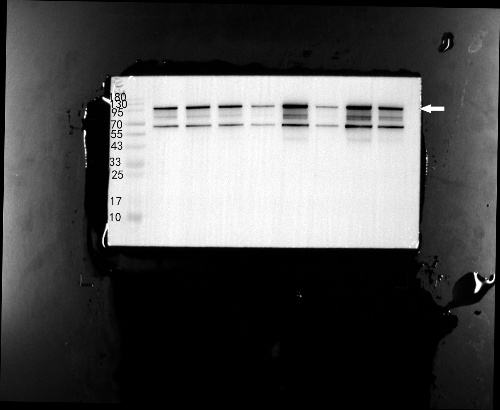
**

**
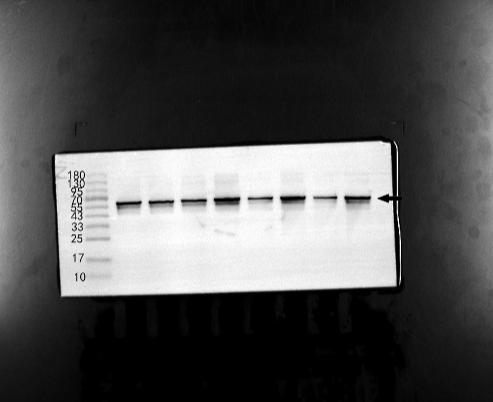

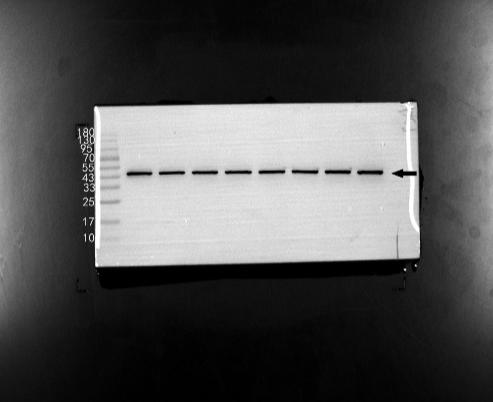
**

**Figure 4f**

HepG2

**
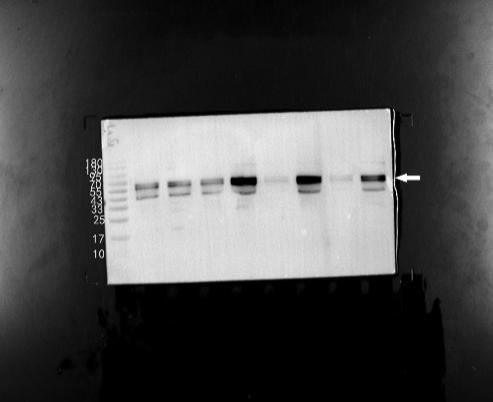

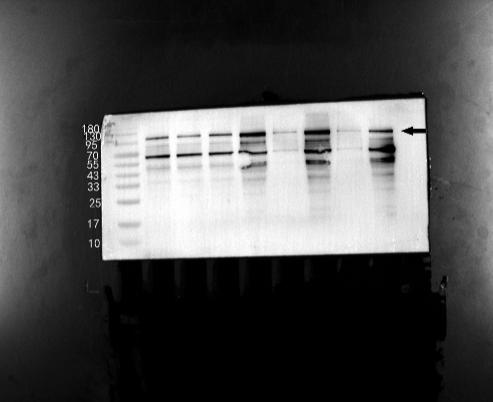
**

**
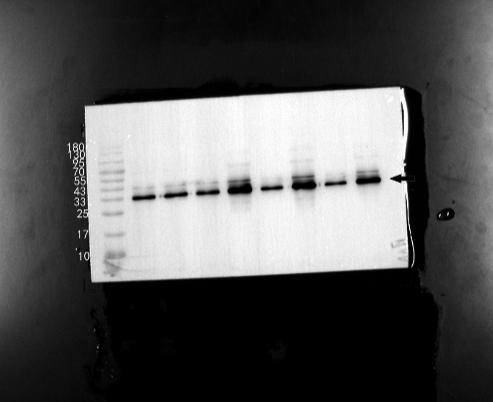

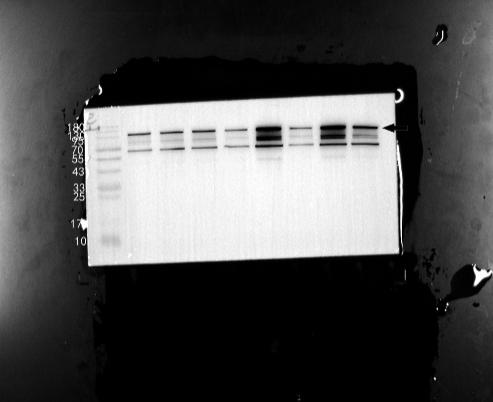
**

**
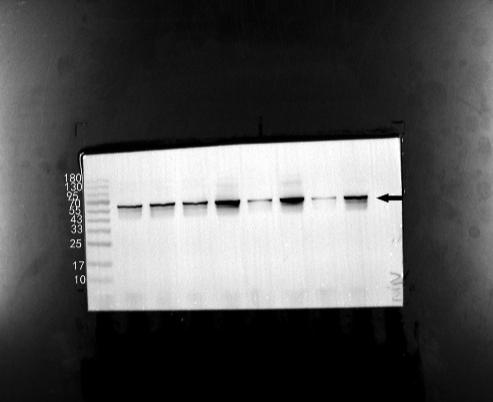

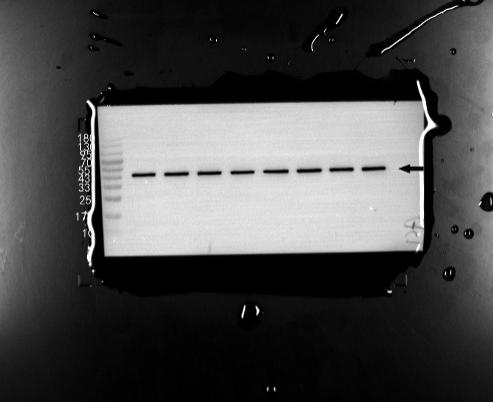
**

**Figure 4i**

Huh7


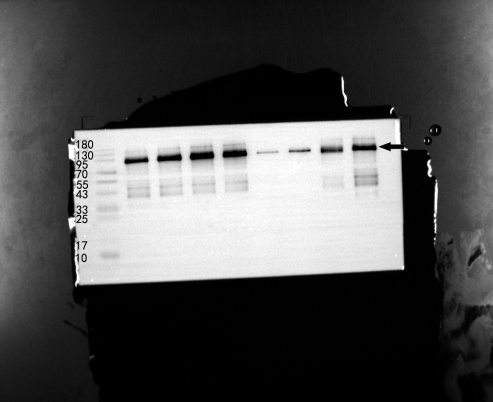

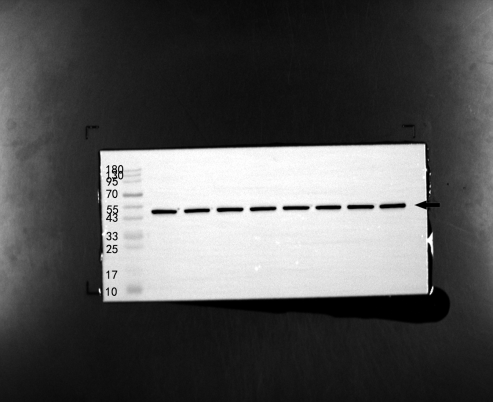


HepG2


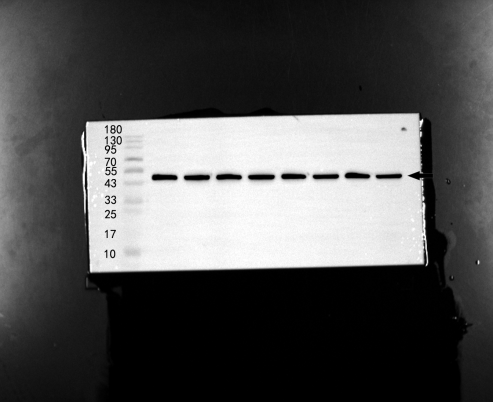

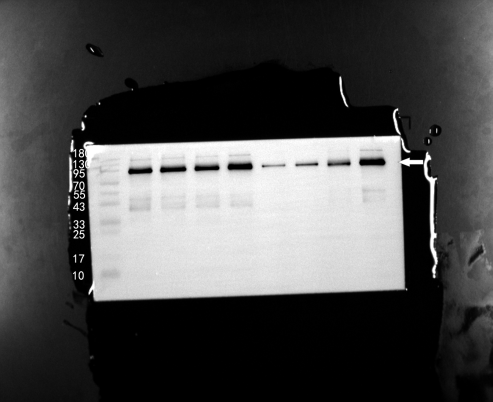


**Figure 4j**

Huh7


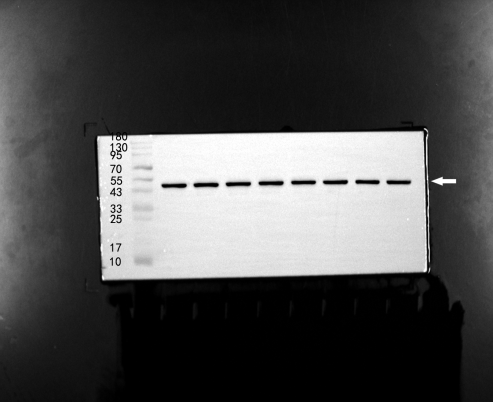

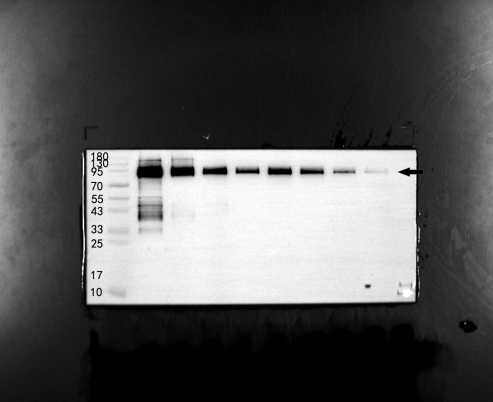


HepG2


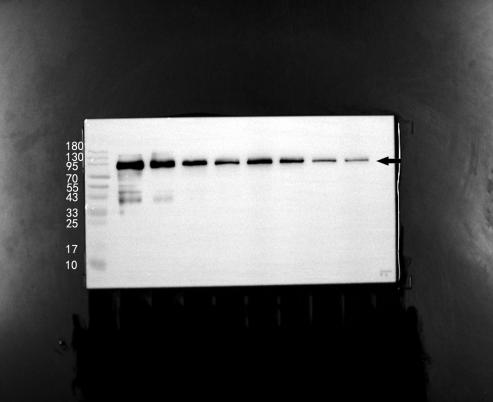

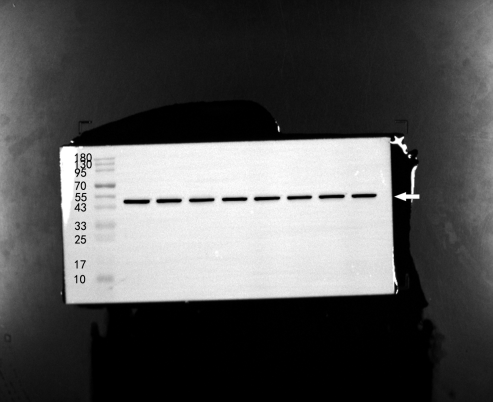


**Figure 5a**

Huh7


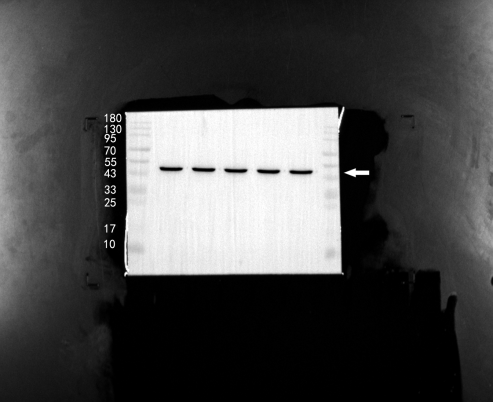

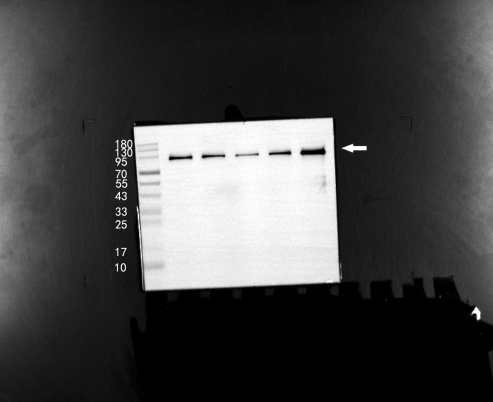

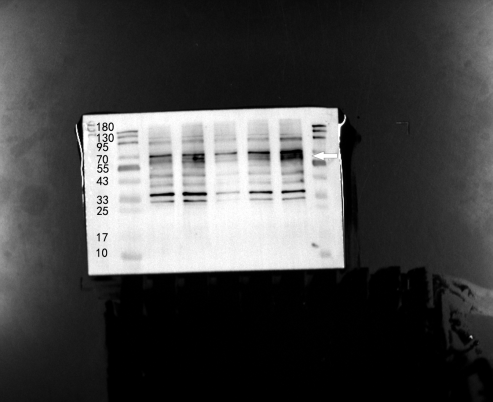

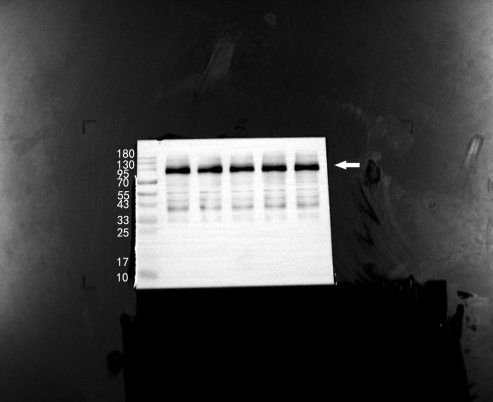

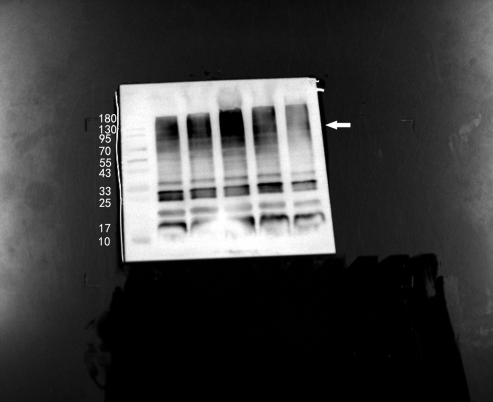


HepG2


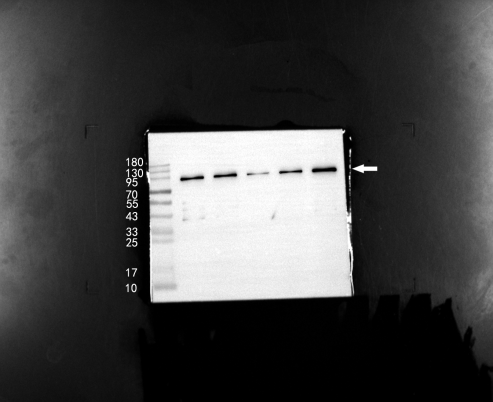

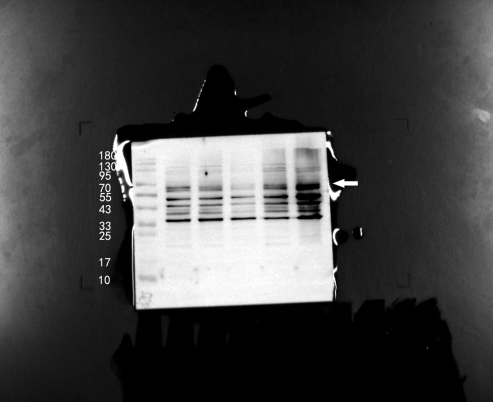

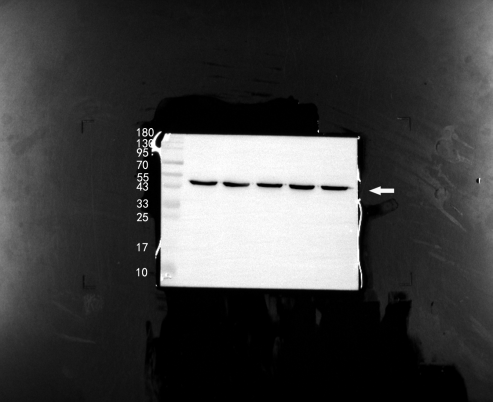

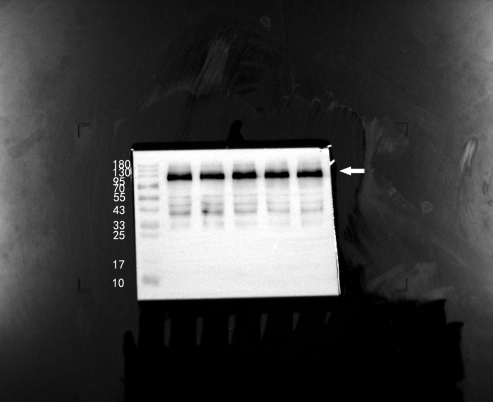

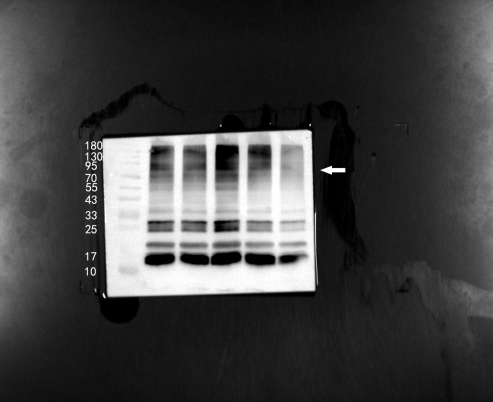


**Figure 5b**

Huh7 ITCH


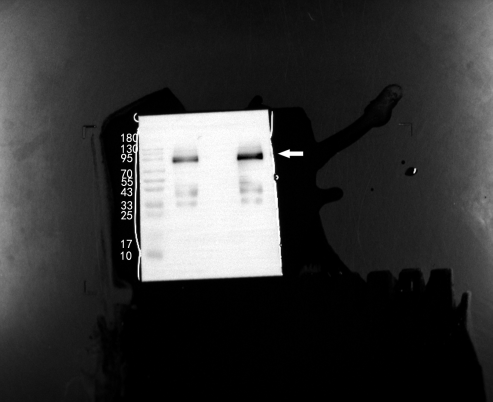

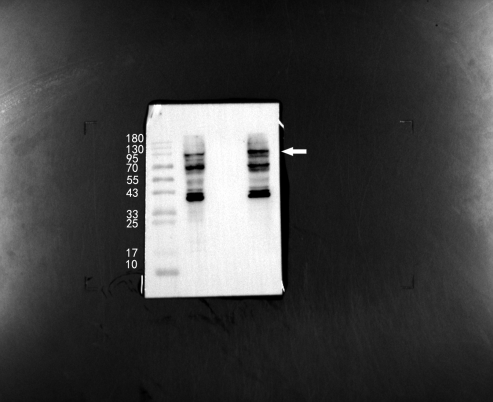


HepG2 ITCH


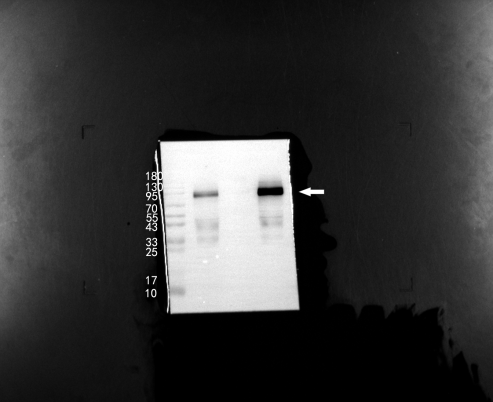

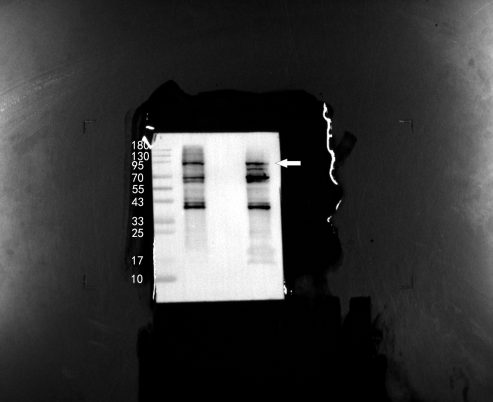


Huh7 ITGB3


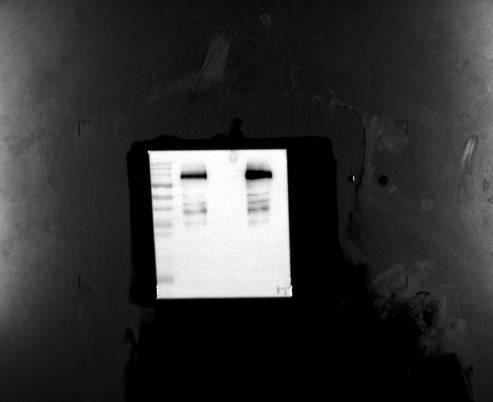

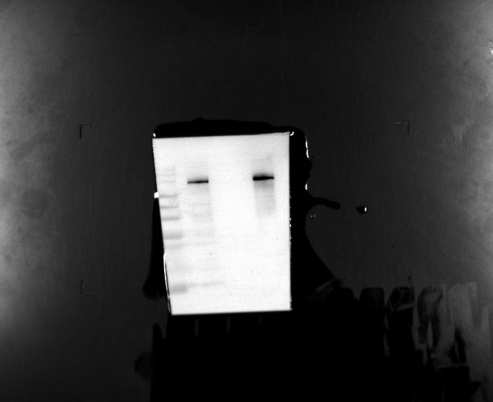


HepG2 ITGB3


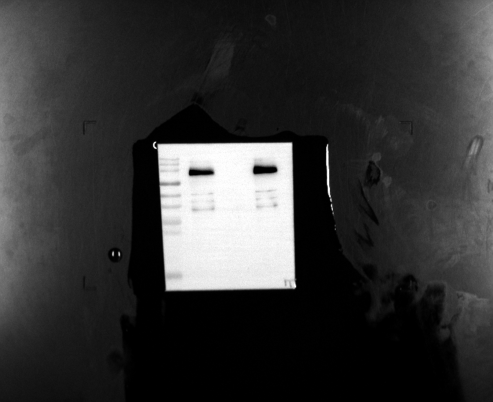

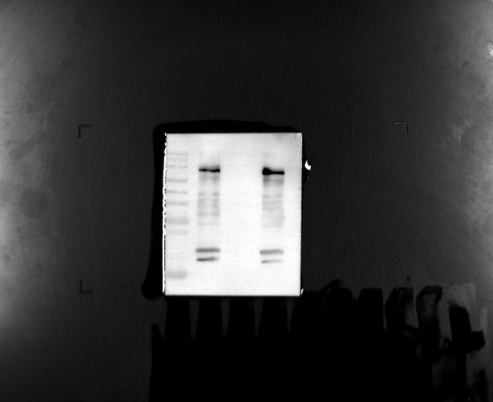


**Figure 5d**

Huh7 ITCH


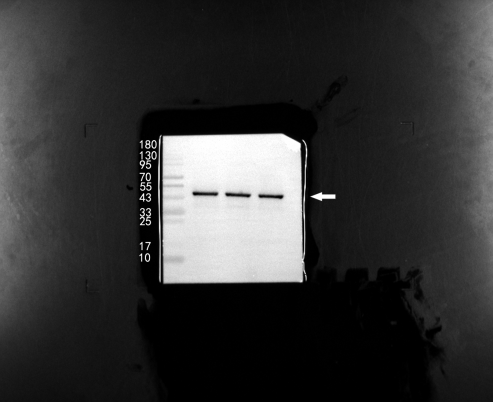

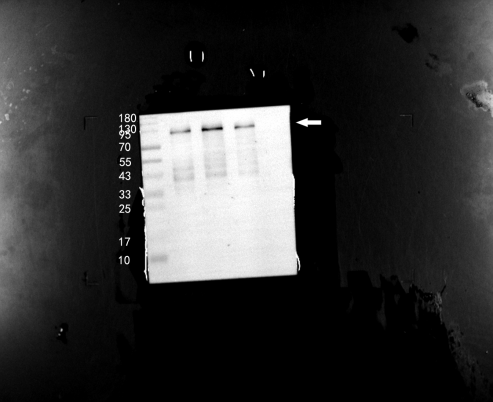

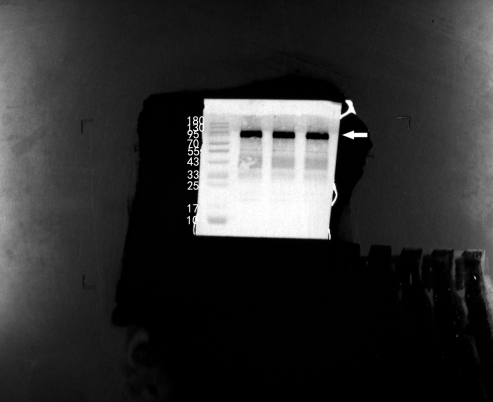

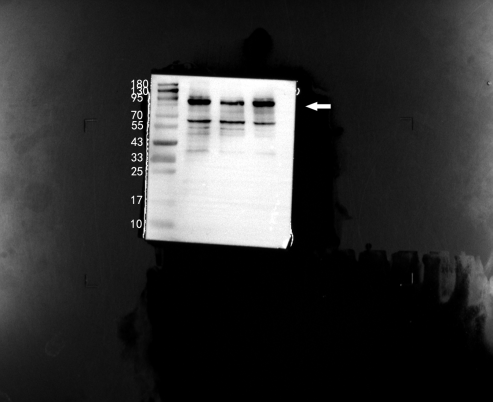


HepG2 ITCH

**
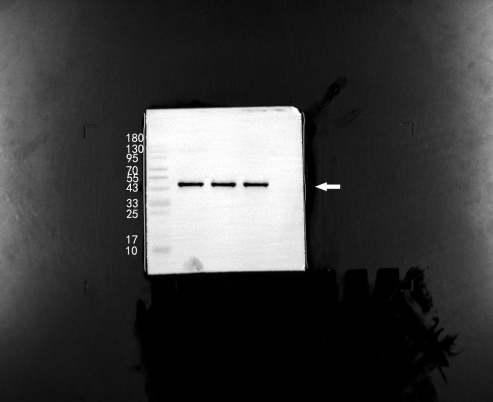

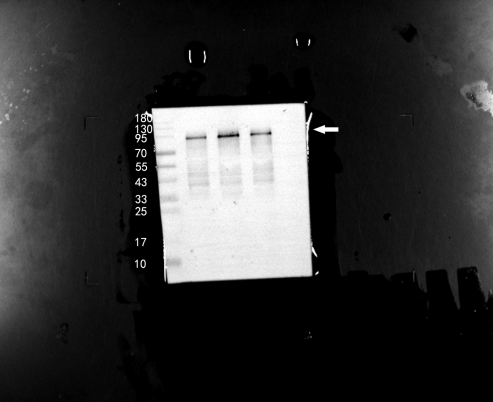
**
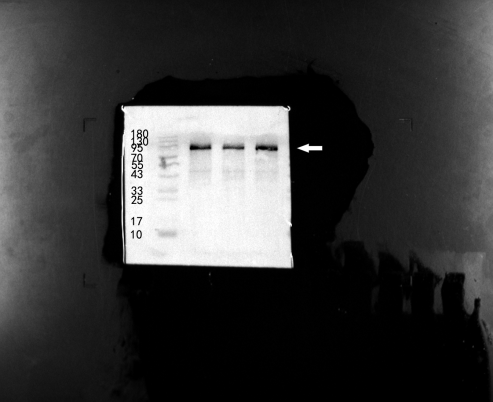

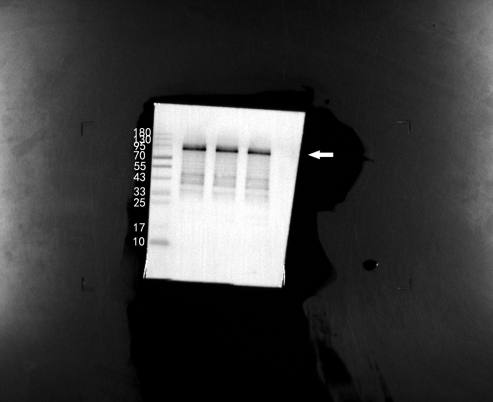


Huh7 ITGB3


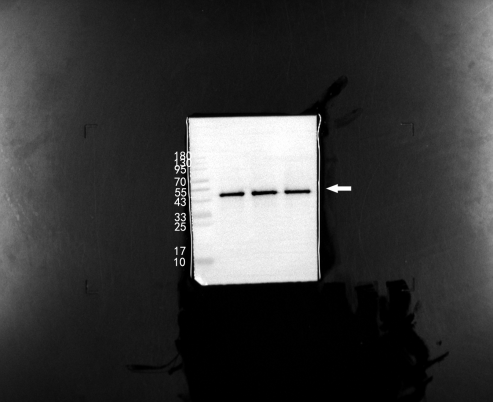


HepG2 ITGB3

**Figure 6c**
