# Supplementary material for: SERPINA1 drives TACE resistance in hepatocellular carcinoma by competitively binding ITGB3 to block ITCH-mediated ubiquitination and degradation
Source: Cell Oncol (Dordr). 2025 Dec 30;49(1):11. doi: 10.1007/s13402-025-01155-5 (PMC12753576; doi:10.1007/s13402-025-01155-5)
Supplement: Supplementary file 2 — Supplementary Material 2 [file 13402_2025_1155_MOESM2_ESM.docx]

**Supplementary Figures and tables**

**SERPINA1 drives TACE resistance in hepatocellular carcinoma by competitively binding ITGB3 to block ITCH-mediated ubiquitination and degradation**

Liou Zhang^1^**^†^**, Xiaoxi Bai^2^**^†^**, Mingyang Du^1^**^†^**, Wenyue Dou^3^, Ziwen Xie^3^, Jie Liu^4*^ and Yang Hou^3*^

^1^Department of Interventional Radiology, Shengjing Hospital of China Medical University, Shenyang, Liaoning Province, 110004, China.

^2^Department of Ultrasound, Shengjing Hospital of China Medical University, Shenyang, Liaoning Province, 110004, China.

^3^Department of Radiology, Shengjing Hospital of China Medical University, Shenyang, Liaoning Province, 110004, China.

^4^Translational Research Experiment Department, Science Experiment Center, China Medical University, Shenyang, Liaoning Province, 110122, China.

**^†^**Liou Zhang, Xiaoxi Bai and Mingyang Du shared co-first authorship.

**^*^Correspondence:** Jie Liu: jieliu@cmu.edu.cn; Yang Hou: houyang1973@163.com.

Table S1. Sequences of the primers

|  | Forward primer (5’ -3’ ) | Reverse primer (5’ -3’ ) |
| --- | --- | --- |
| SERPINA1 | ATGCTGCCCAGAAGACAGATA | CTGAAGGCGAACTCAGCCA |
| HIF1α | GAACGTCGAAAAGAAAAGTCTCG | CCTTATCAAGATGCGAACTCACA |
| ITGB3 | GTGACCTGAAGGAGAATCTGC | CCGGAGTGCAATCCTCTGG |
| β-actin | CATGTACGTTGCTATCCAGGC | CTCCTTAATGTCACGCACGAT |

Table S2. Antibody information for Western blot and immunohistochemistry

| Name | Manufacturer | Catalog Number |
| --- | --- | --- |
| HRP, Goat Anti-Mouse IgG | Abbkin | A21010 |
| HRP, Goat Anti-Rabbit IgG | Abbkin | A21020 |
| Beta Actin Monoclonal antibody | Proteintech | 66009-1-Ig |
| PCNA Polyclonal antibody | Proteintech | 10205-2-AP |
| Alpha 1 Antitrypsin Polyclonal antibody | Proteintech | 16382-1-AP |
| HIF-1 alpha Polyclonal antibody | Proteintech | 20960-1-AP |
| CD61 / Integrin beta 3 Polyclonal antibody | Proteintech | 18309-1-AP |
| Pierce® GST Protein Interaction Pull-Down Kit | Thermo Fisher | 21516 |
| GST Tag Polyclonal antibody | Proteintech | 10000-0-AP |
| IPKine™ HRP, Goat Anti-Rabbit IgG HCS | Abbkin | A25222 |
| IPKine™ HRP, Mouse Anti-Rabbit IgG HCS | Abbkin | A25122 |
| HA tag Polyclonal antibody | Proteintech | 51064-2-AP |
| DYKDDDDK tag Polyclonal antibody (Binds to FLAG® tag epitope) | Proteintech | 20543-1-AP |
| MYC tag Polyclonal antibody | Proteintech | 16286-1-AP |
| ITCH Polyclonal antibody | Proteintech | 20920-1-AP |
| E-cadherin Polyclonal antibody | Proteintech | 20874-1-AP |
| Vimentin Monoclonal antibody | Proteintech | 60330-1-Ig |
| CD61 / Integrin beta 3 Monoclonal antibody | Proteintech | 66952-1-Ig |

Table S3. The siRNA target sequences

| Name | Sequences |
| --- | --- |
| ITGB3-siRNA-1-sense | 5’-UGUUGAUGCUUAUGGGAAATT-3’ |
| ITGB3-siRNA-1-antisense | 5’-UUUCCCAUAAGCAUCAACATT-3’ |
| ITGB3-siRNA-2-sense | 5’-CCUAUAAGAAUGAGGAUGATT-3’ |
| ITGB3-siRNA-2-antisense | 5’-UCAUCCUCAUUCUUAUAGGTT-3’ |
| ITGB3-siRNA-3-sense | 5’-GAGAAUCUGCUGAAGGAUATT-3’ |
| ITGB3-siRNA-3-antisense | 5’-UAUCCUUCAGCAGAUUCUCTT-3’ |

a b


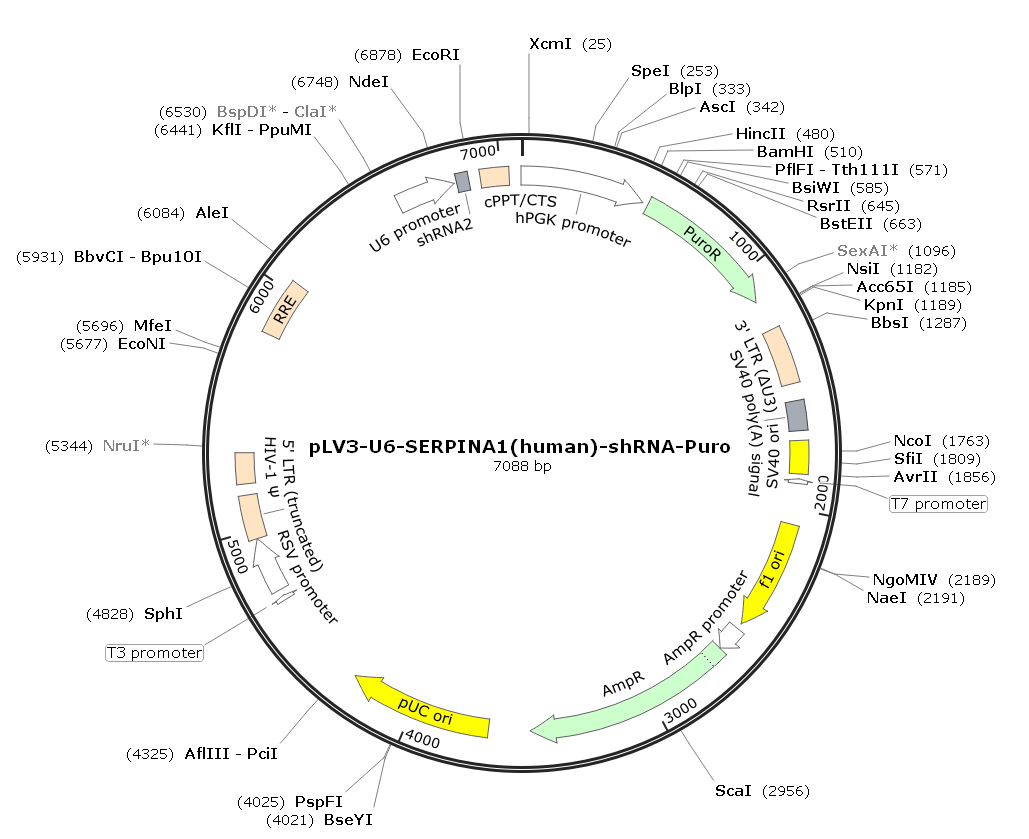

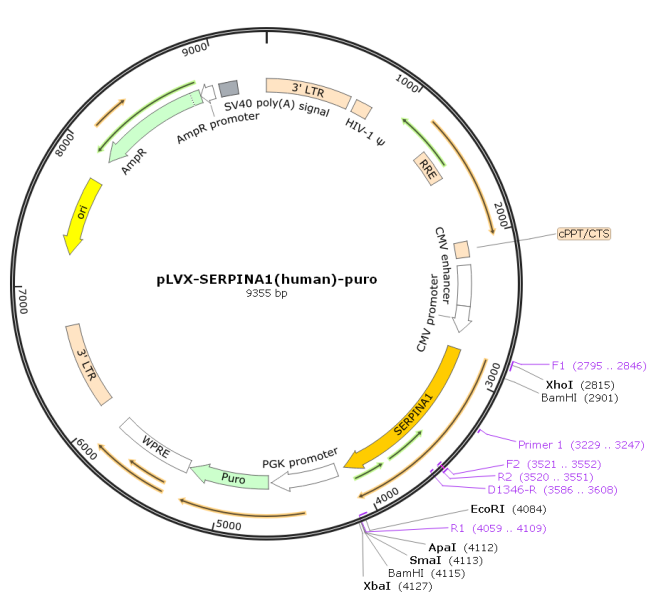


Fig. S1 Schematic diagrams of lentiviral vector plasmids

(a)Lentiviral vector plasmid for SERPINA1 knockdown. (b) Lentiviral vector plasmid for SERPINA1 overexpression.


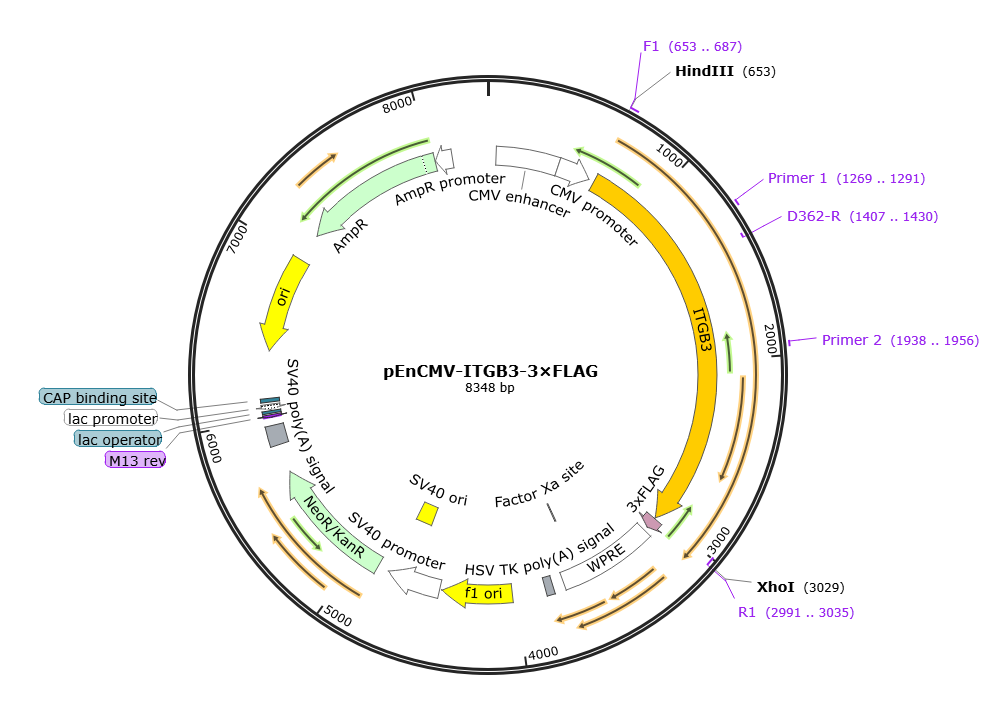


Fig. S2 ITGB3 Overexpression Plasmid Map


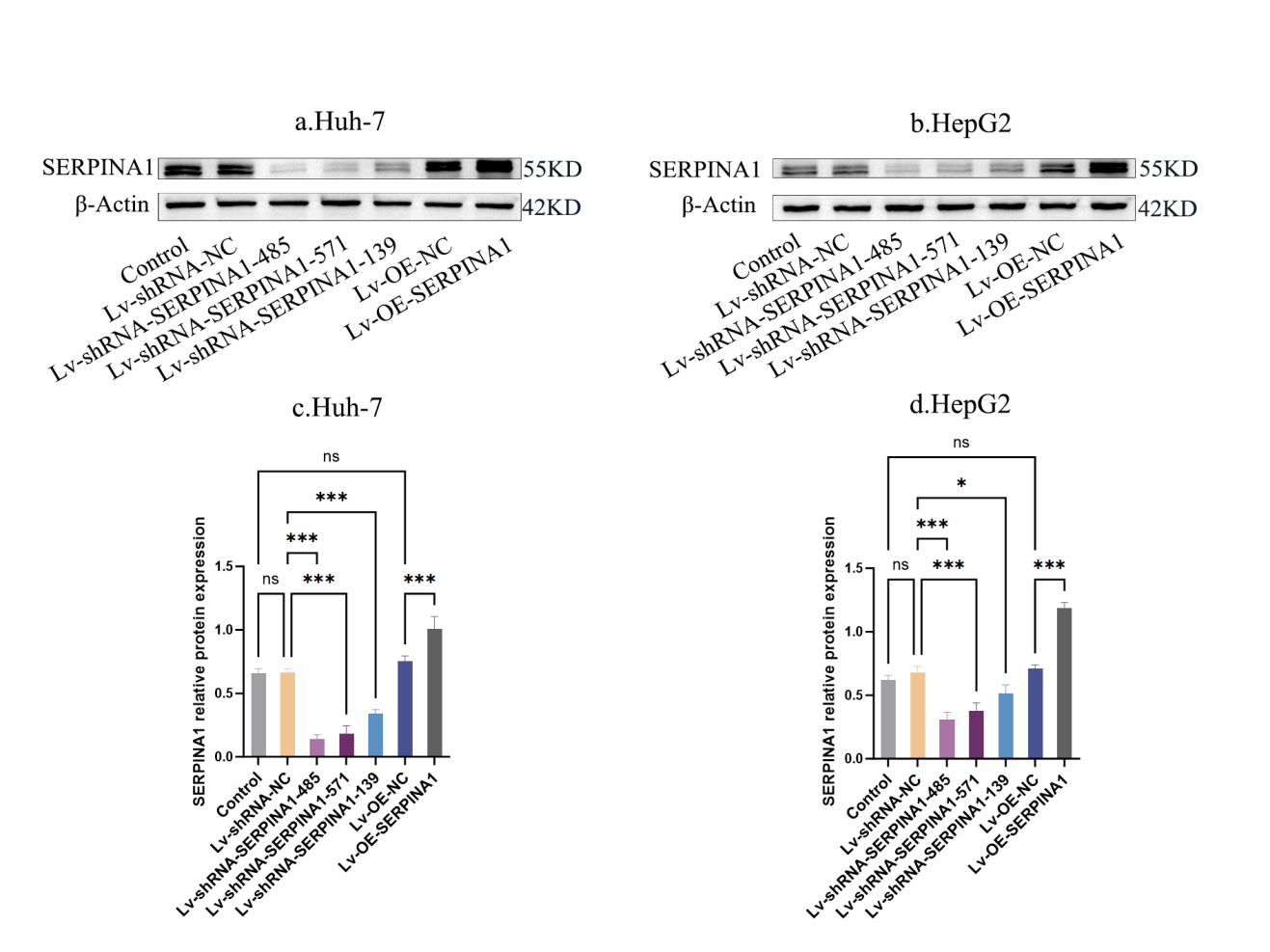


Fig. S3 Screening of SERPINA1 expression in lentivirus-transfected hepatocellular carcinoma cell lines by Western blot.

(a, c)Huh-7 cells; (b, d)HepG2 cells. Lv-shRNA-SERPINA1-485 effectively downregulated SERPINA1 expression in different HCC cell lines, whereas Lv-OE-SERPINA1 upregulated SERPINA1 expression. **P* < 0.05, ****P* < 0.001

a

b

c

Fig. S4 IP-MS spectra.

d

(a)Input-MS detection of SERPINA1 peptide VFSNGADLSGVTEEAPLK. (b)Input-MS detection of ITGB3 peptide SFTIKPVGFK. (c)IP-MS detection of SERPINA1 peptide VFSNGADLSGVTEEAPLK. (d)IP-MS detection of ITGB3 peptide SFTIKPVGFK.


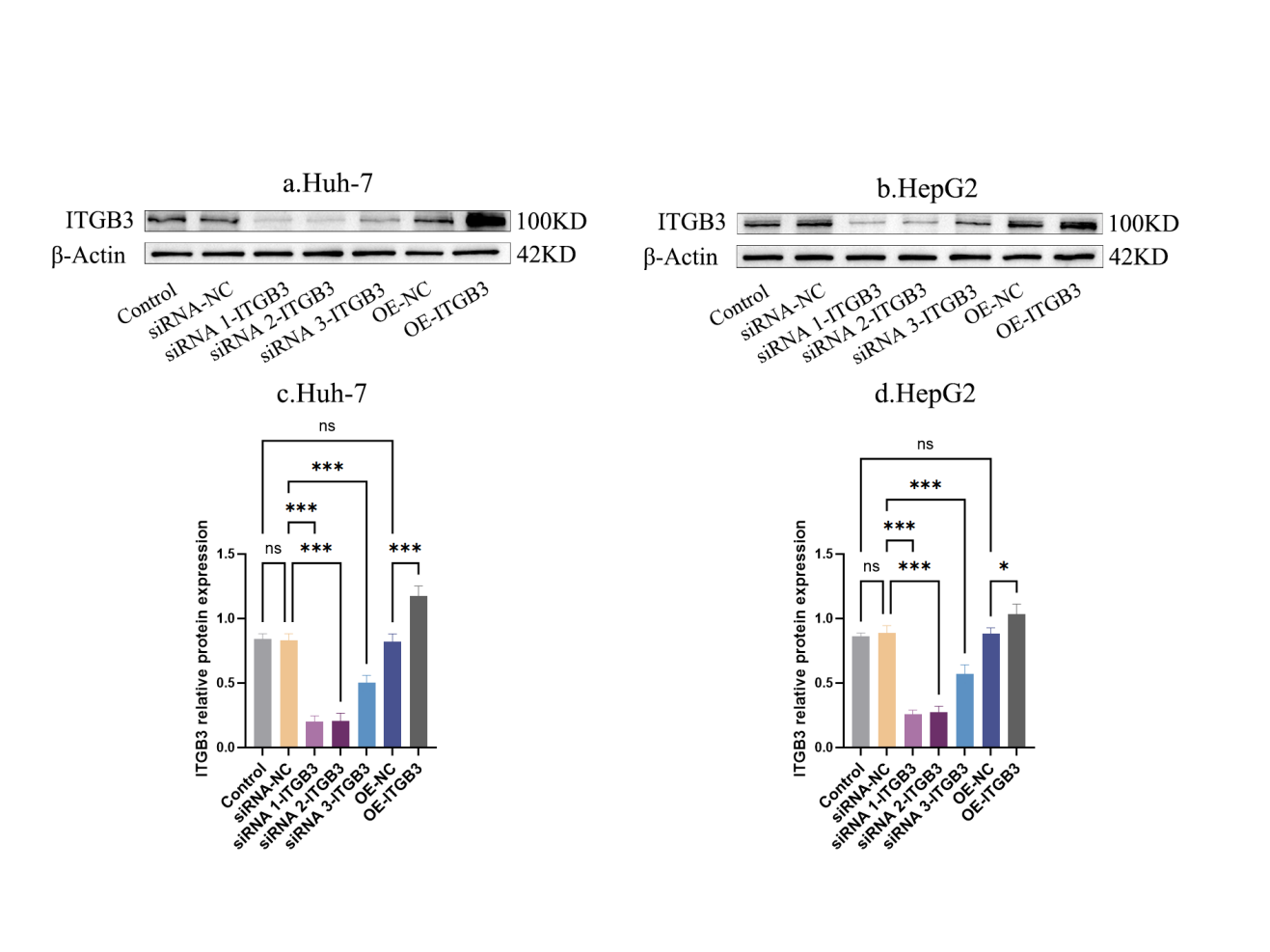


Fig. S5 Validation of transfection efficiency for plasmid and siRNA.

(a, c) Huh-7 cells; (b, d) HepG2 cells. Transfection with the ITGB3 overexpression plasmid significantly upregulated ITGB3 expression in different HCC cell lines, whereas transfection with siRNA effectively downregulated ITGB3 expression. **P* < 0.05, ****P* < 0.001
